# Targeting the SOX2/CDP protein complex with a peptide suppresses the malignant progression of esophageal squamous cell carcinoma

**DOI:** 10.1038/s41420-023-01693-7

**Published:** 2023-10-27

**Authors:** Yunyun Chen, Kun Zhang, Rui Zhang, Zhuo Wang, Liang Yang, Tingting Zhao, Shihui Zhang, Yong Lin, Hongzhou Zhao, Yongpan Liu, Yuxuan Wei, Yijian Zhou, Jiaying Zhang, Xianzong Ye, Jing Zhao, Xinxin Li, Jianwen Que, Songlin Shi, Kuancan Liu

**Affiliations:** 1 Central Laboratory, Xiang’an Hospital of Xiamen University, School of Medicine, Xiamen University, Xiamen, Fujian 361102 P. R. China; 2 School of Medicine, Xiamen University, Xiamen, Fujian 361102 P. R. China; 3 Department of General Surgery, Fuzhou First General Hospital affiliated with Fujian Medical University, Fuzhou, Fujian 350009 P. R. China; 4 Department of Laboratory Medicine, The Second Hospital of Fuzhou, Fuzhou, Fujian 350007 P. R. China; 5 Westlake University, Hangzhou, Zhejiang 310024 P. R. China; 6 Centre for Translational Stem Cell Biology, School of Biomedical Sciences, The University of Hong Kong, Pokfulam, Hong Kong SAR, 999077 P. R. China; 7 Science and Technology Service Center, Fujian Health College, Fuzhou, Fujian 350101 P. R. China; 8 School of Life Science, Xiamen University, Xiamen, Fujian 361102 P. R. China; 9 Department of Pathology, 900 Hospital of the Joint Logistics Team (Dongfang Hospital, Xiamen University), Fuzhou, Fujian 350025 P. R. China; 10 Department of Medicine, Columbia University Medical Center, New York, NY 10032 USA; 11 School of Life Science, Nanchang Normal University, Nanchang, Jiangxi 330032 P. R. China

**Keywords:** Drug development, Targeted therapies

## Abstract

Emerging evidence indicates that SOX2 is an oncogene for esophageal squamous cell carcinoma (ESCC). However, direct targeting of SOX2 is not feasible given that this transcription factor plays important roles in the maintenance of tissues such as the brain. Here, we identified CDP (Homeobox protein cut-like 1 or CASP) as a unique SOX2 binding partner enriched in ESCC with Duolink proximity ligation assay, bimolecular fluorescence complementation (BiFc) and immunoprecipitation. We then screened a peptide aptamer library using BiFc and immunoprecipitation and identified several peptide aptamers, including P58, that blocked the CDP/SOX2 interaction, leading to the inhibition of ESCC progress in vitro and in vivo. Upon administration, synthetic peptide P58, containing the YGRKKRRQRRR cell-penetrating peptide and the fluorophore TAMRA, also blocked the growth and metastasis of ESCC in both mice and zebrafish. Therefore, targeting the SOX2 binding partner CDP with peptide P58 offers an alternative avenue to treat ESCC with increased SOX2 levels.

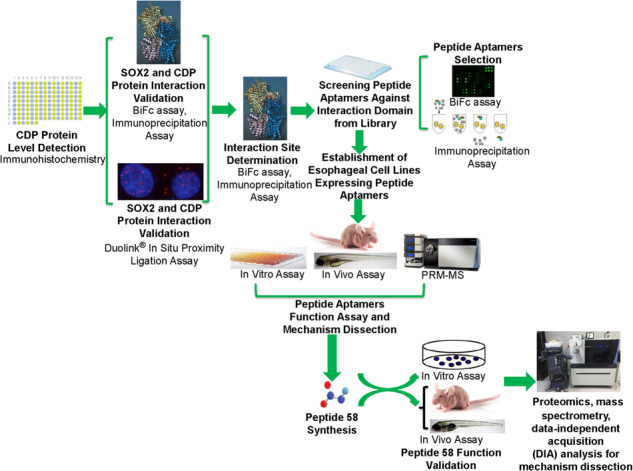

## Introduction

Esophageal squamous cell carcinoma (ESCC) is the major esophageal malignancy in developing countries and is responsible for 90% of esophageal cancer deaths in China. The etiology of ESCC involves both environmental and genetic components, such as drinking alcohol and family history of ESCC [1], and viral infection [2]. Genetic studies have revealed that approximately 30% of ESCCs exhibit amplification of the transcription factor SOX2 [3, 4].

SOX2 plays critical roles in organ development and stem cell maintenance [5–9]. Emerging evidence has revealed that SOX2 protein levels are closely linked with tumor-associated processes, including tumorigenesis [3, 4, 10, 11], tumor maintenance [12, 13], invasion, and metastasis [14–16]. SOX2 can also exert its roles by interacting with various proteins, such as β-catenin [17], PARP1(Poly(ADP-Ribose) polymerase 1) [18]. Interestingly, SOX2 also interacts with Chd7 (chromodomain helicase DNA binding protein 7) and the transcription factor CDP [19]. CDP encoded by CUX1 (also known as CUTL1) is involved in many cellular activities, including development and tumorigenesis [20–23]. CDP protein also affects the activities of multiple signaling pathways, including WNT/β-catenin, TNF, JAK/STAT, MAPK/ERK, Hedgehog, and PI3K/Akt [24]. Therefore, we investigated the role of the SOX2/CDP interaction in ESCC, which may provide potential strategies for ESCC treatment. In the present study, we show that CDP interacted with SOX2 in ESCC tissues and cell lines. We then identified a peptide aptamer (P58) that specifically blocked the CDP/SOX2 interaction by screening a peptide aptamer library. Further investigation showed that the P58 aptamer inhibited ESCC cell proliferation, migration, and invasion in vitro. Upon injection, P58 reduced tumor growth in mouse and metastasis in zebrafish models. Intriguingly, the synthetic form of peptide 58, in which the conformation was constrained by a disulfide bond, was also found to suppress tumor growth in nude mice and metastasis in zebrafish. Incubation of ESCC cells with synthetic peptide 58 led to the differential expression of genes associated with the progression of ESCC.

## Results

### CDP is enriched in ESCC clinical tissues and cells and is associated with a poor prognosis

**Fig. 1:**
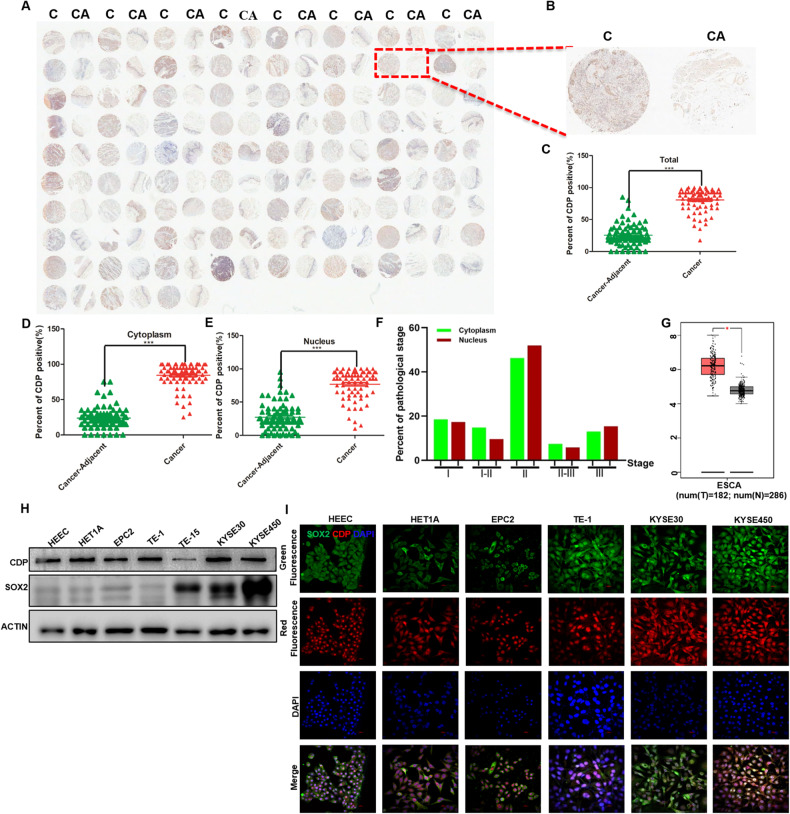
CDP protein levels are markedly increased in ESCC clinical samples and cell lines. **A** High levels of CDP protein in ESCC clinical samples, shown by immunostaining on a tissue chip. C: Cancer; CA: Cancer adjacent normal tissues. **B** Representative figure showing CDP protein expression in an ESCC sample and its matched normal tissue. **C** Significantly higher levels of CDP protein in ESCC tumor tissues (*n* = 75, *p* < 0.001). **D** Significantly higher levels of CDP protein in the cytoplasm of ESCC tumor tissues (*n* = 75, *p* < 0.001). **E** Significantly higher levels of CDP protein in the nuclei of ESCC tumor tissues (*n* = 75, *p* < 0.001). **F** Percent of pathological stage in ESCC clinical samples when the correlation index of CDP and SOX2 proteins reached 1. **G** CUX1 mRNA levels in ESCA tumor samples, determined in 182 ESCA tissues and 286 normal tissue samples using GEPIA (*p* < 0.05). **H** Abundant expression of both CDP and SOX2 proteins in ESCC cells. **I** Colocalization of SOX2 and CDP proteins in multiple esophageal cell lines. Scale bar: 20 μm. * *p* < 0.05, ** *p* < 0.01, *** *p* < 0.001 vs. control. The data represent the means ± SDs.

CDP protein levels were measured on an ESCC tissue chip containing 75 pairs of ESCC specimens and their matched adjacent normal tissues (Fig. 1A); a representative enlargement of one case stained with the anti-CDP antibody is shown in Fig. 1B. Moreover, CDP protein levels were significantly elevated in cancer tissues compared with their normal counterparts (Fig. 1C, *p* < 0.001), with CDP observed uniformly in the cytoplasm and nucleus (Fig. 1D, E, *p* < 0.001). The clinicopathological characteristics of the 75 ESCC patients showed that most were at stage N (61.3%) and histopathological grade II (45.3%) (Supplied Table 1), indicating that high CDP levels in these patients were associated with poorer prognosis. More importantly, we analyzed the correlation index of CDP and SOX2 proteins after the combination analysis of their expression levels in these ESCC clinical samples [25], and we found that correlation index of these two proteins reached 1 in 72% of cytoplasm and 69.3% of nucleus in clinical samples, and more clinical samples with correlation index at 1 occurred in the advanced stage of ESCC, such as stage II, II-III and III (Fig. 1F).

On the other hand, CUX1 mRNA expression was analyzed using the GEPIA tool (http://gepia.cancer-pku.cn/index.html). The results showed that CUX1 expression was higher in 182 ESCA clinical samples than in 286 matched Cancer Genome Atlas (TCGA) normal and GTEx data (Fig. 1G, *p* < 0.05). The protein expression levels of CDP and SOX2 in multiple cell lines were also detected with western blotting, and the results revealed that both CDP and SOX2 proteins were abundantly expressed in KYSE30 and KYSE450 cells (Fig. 1H). In addition, we also measured CDP protein levels in HEEC cells (normal esophageal epithelial cells), HET1A cells, EPC2 cells, and ESCC cell lines using immunofluorescence. As shown in Fig. 1I, the two proteins were expressed and colocalized in HEEC, HET1A, EPC2, TE-1, KYSE30, and KYSE450 cells; however, higher levels of these two proteins were observed in ESCC cells.

### Interaction interface between SOX2 and CDP protein is confirmed after mutation analysis

**Fig. 2:**
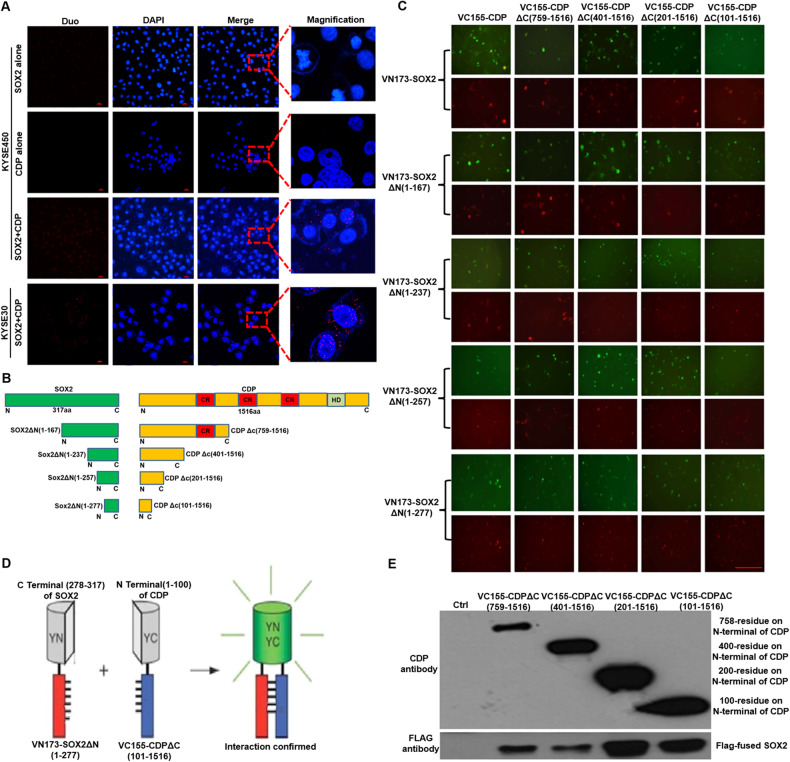
The interaction interface between SOX2 and CDP protein is determined by Duolink PLA, BiFc and immunoprecipitation assays. **A** Interaction between CDP and SOX2 protein was analyzed by Duolink PLA in KYSE450 cells and KYSE30 cells. PLA signals were detected by fluorescence microscopy and determined by discrete red spots. A single SOX2 antibody alone or a single CDP antibody alone in KYSE450 cells was used as a negative control. **B** Multiple constructs expressing full-length CDP protein and SOX2 protein or their respective truncated mutants were used for the BiFc assay. **C** Interactions between CDP protein, SOX2 protein, and their respective mutants were verified by BiFc. Scale bar: 50 μm. **D** The interacting interface on the CDP protein and SOX2 protein were explored with a cotransfection BiFc assay. Note that the interaction between the 1-100 N-terminal CDP sequence and the 278-317 sequence of SOX2 was verified by BiFc assay. **E** Confirmation of the interaction between CDP protein mutants and SOX2 protein by immunoprecipitation.

To detect the endogenous interaction between CDP and SOX2 protein under normal physiological conditions, a Duolink PLA system was applied to detect their interaction in KYSE450 and KYSE30 cells. The in-situ interaction between these two proteins, which was reflected by the PLA signals, was confirmed by fluorescence microscopy as the occurrence of discrete red spots (Fig. 2A). To pinpoint the CDP-SOX2 interaction interface, we produced a series of constructs comprising the full-length or truncated coding sequences for SOX2 and CDP with primers (Fig. 2B; supplied Fig. 1A, B, supplied Table 2). Cotransfection of 25 construct assemblies into HEK293T cells resulted in green fluorescence, indicating an interaction between the two proteins. Notably, we also observed green fluorescence after the cotransfection of VN173-SOX2ΔN (1-277) with VC155-CDPΔC (101-1516), suggesting that the interaction interface was located between residues 278-317 at the SOX2 C-terminus and residues 1-100 at the CDP N-terminus (Fig. 2C, D).

To further verify the interaction between the 1-100-residue sequence of CDP and the full-length SOX2 protein, VC155 constructs for the expression of truncated CDP and VN173-SOX2 for the expression of the FLAG-fused SOX2 protein were obtained and cotransfected into cells, followed by immunoprecipitation with anti-CDP and anti-FLAG antibodies to detect CDP and the FLAG-fused SOX2 protein, respectively. As shown in Fig. 2E, SOX2 interacted with residues 1-758, residues 1-400, residues 1-200, and residues 1-100 at the CDP N-terminus.

### Identification of peptide aptamer P58 targeting the interaction interface on CDP

**Fig. 3:**
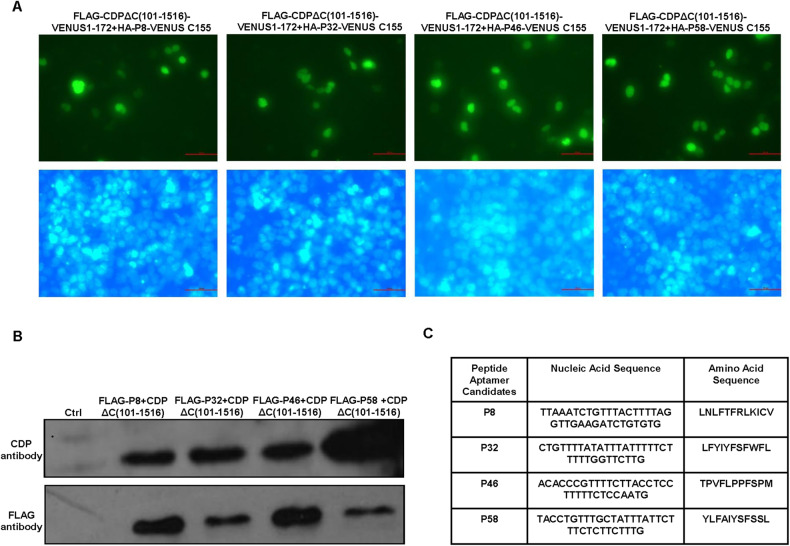
Peptide aptamer candidates targeting the interaction interface on the N-terminus of CDP protein are screened from a peptide aptamer library. **A** Four candidate peptide aptamers targeting the interaction interface on CDP protein were screened using BiFc after cotransfection of pBiFc-VN173-CDPΔC (101-1516) with plasmids from the pBiFc-VC155-TrxA-peptide-TrxA library. Scale bar: 50 μm. **B** Interactions between Flag-fused four candidate peptide aptamers and the 100-residue N-terminal of CDP protein was shown by immunoprecipitation after cotransfection of pCMV-Tag2B-peptide and pBiFcVC155-CDPΔC (101-1516), respectively. **C** Nucleotide sequences coding four candidate peptide aptamers and amino acid sequences.

To identify a peptide aptamer recognizing the interaction interface on CDP protein (CDPΔC101-1516), pBiFc-VN173-CDPΔC (101-1516) (supplied Fig. 1C), which could drive the expression of this specific interface with VENUS 1-172 and Flag tag, was produced using the primers listed in supplied Table 3. Unbiased screening of the pBiFc-VC155-TrxA-peptide-TrxA library, a library for fused expression of constrained peptide aptamers with VENUS C155 and HA TAG, was then performed using the BiFc assay, which had been used for screening specific peptide aptamers against SOX2 protein [25]. After the cotransfection of each pBiFc-VC155-TrxA-peptide-TrxA from the library with pBiFc-VN173-CDPΔC (101-1516), we found that the peptide aptamers P8, P32, P46, and P58 could interact with CDPΔC (101-1516) based on the occurrence of green fluorescence (Fig. 3A).

To verify these results described above, four pCMV-Tag2B-peptide constructs, which drive the expression of P8, P32, P46, and P58 peptide aptamers fused with the FLAG tag, were produced (Supplied Fig. 1D). pBiFcVC155-CDPΔC (101-1516), which was previously used to express CDPΔC (101-1516) fused with the HA tag and VENUS C155 in the BiFc assay, and each pCMV-Tag2B-peptide construct were cotransfected into cells, followed by immunoprecipitation to confirm interactions between CDPΔC (101-1516) and the four peptide aptamer candidates. As shown in Fig. 3B, interactions between CDPΔC (101-1516) and the peptide aptamers P8, P32, P46, and P58 were confirmed. The nucleotide sequences coding these peptide aptamers and their amino acid sequences are shown in Fig. 3C.

### Expression of the P58 aptamer suppresses multiple malignant processes of ESCC in vitro

To investigate the actions of the peptide aptamers, we used the lentiviral vector pCDH-CMV-Oligo-IRES-GFP-EF1-Puro, which was modified from our previous study [3, 14, 25], to express peptide aptamers P8, P32, P46, and P58 after ligation. After packaging (supplied Fig. 2A), lentiviral infection of KYSE450 and KYSE30 cells, and drug selection, stable infected lines were obtained with pCDH-CMV-peptide aptamer-IRES-GFP-EF1-Puro, and pCDH-CMV-Oligo-IRES-GFP-EF1-Puro served as a control (Supplied Fig. 2B).

**Fig. 4:**
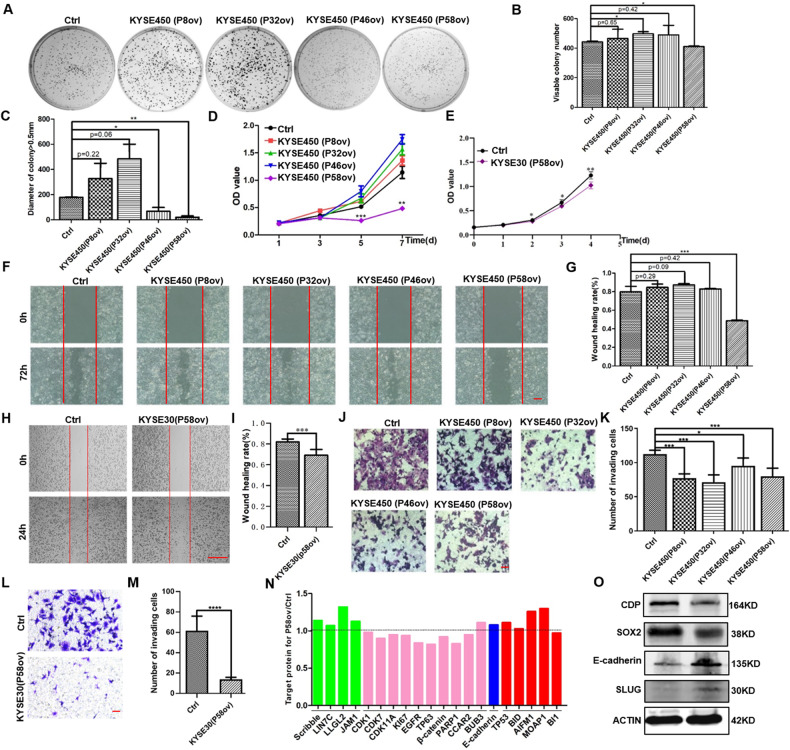
Inhibition of malignant processes in ESCC cells is achieved after the ectopic expression of P58 peptide aptamer. **A**, **B** Ectopic expression of the P58 peptide aptamer reduced colony numbers in KYSE450 cells (*p* < 0.05). **C** Ectopic expression of the P58 peptide aptamer reduced the number of colonies with sizes larger than 0.5 mm in KYSE450 cells (*p* < 0.01 for the P58 peptide aptamer and *p* < 0.05 for the P46 peptide aptamer). **D** The P58 peptide aptamer reduced proliferation in KYSE450 cells, as measured by CCK8 assay (*p* < 0.001 on Day 5; *p* < 0.01 on Day 7). **E** The P58 peptide aptamer reduced proliferation in KYSE30 cells, as measured by CCK8 assay (*p* < 0.05 on Day 3; p < 0.01 on Day 4). **F**, **G** The p58 peptide aptamer inhibited the migration of KYSE450 cells, as shown by the wound-healing assay and statistical analysis (*p* < 0.001). **H**, **I** The p58 peptide aptamer reduced migration in KYSE30 cells, as shown by the wound-healing assay and statistical analysis (*p* < 0.001). **J**, **K** The P58 peptide aptamer suppressed invasion in KYSE450 cells, as shown by the transwell assay and statistical analysis (*p* < 0.001 for the P8, P32, and P58 peptide aptamers, while *p* < 0.05 for the P46 peptide aptamer). **L**, **M** The p58 peptide aptamer reduced invasion in KYSE30 cells, as shown by the transwell assay and statistical analysis (*p* < 0.0001). **N** PRM-MS proteomic analysis revealed changes in protein levels in KYSE450 cells upon the ectopic expression of P58 peptide aptamer. Note that a value >1 indicates higher expression in comparison with control cells, and a value <1 indicates lower expression in comparison with control cells. **O** Changed levels of specific pivotal proteins in KYSE450 cells after the ectopic expression of P58 peptide aptamer. * *p* < 0.05, ** *p* < 0.01, *** *p* < 0.001 vs. control. The data represent the means ± SDs.

Both KYSE450 and KYSE30 lines showed elevated expression of CDP and SOX2 (Fig. 1G), and we investigated whether the ectopic expression of the peptide aptamers affected the formation of colonies from single cells. Significant differences in colony-forming ability were observed after the ectopic expression of the P32 and P58 peptide aptamers (*p* < 0.05) (Fig. 4A, B). Moreover, the number of larger colonies (>0.5 mm) formed by KYSE450 cells was significantly reduced after the ectopic expression of P46 and P58 peptide aptamers in comparison with controls (Fig. 4C, *p* < 0.05 for P46 and *p* < 0.01 for P58). In addition, the ectopic expression of the P58 peptide aptamer resulted in significantly lower proliferation in KYSE450 cells, as shown by a CCK8 assay on the seventh day (Fig. 4D, *p* < 0.01). Similarly, ectopic expression of the P58 peptide aptamer also significantly inhibited KYSE30 cell proliferation on the fourth day (Fig. 4E, *p* < 0.01).

We next investigated the influence of peptide aptamer expression on cell migration and invasion. Ectopic expression of the P58 peptide aptamer significantly reduced migration in KYSE450 cells, as shown by the wound-healing assay, and reduced the healing index from 79.9% (control) to 48.7% (Fig. 4F, G, *p* < 0.001). Similarly, reduced migration was also observed in KYSE30 cells after the ectopic expression of the P58 peptide aptamer (Fig. 4H), with a reduction in the healing index from 82% (control) to 69.2% (Fig. 4I, *p* < 0.001).

Ectopic expression of all four peptide aptamers also blocked the invasion of KYSE450 cells, as shown by transwell assays (Fig. 4J). As shown in Fig. 4K, invasion was reduced to 68.7% for P8 (*p* < 0.001), 63.4% for P32 (*p* < 0.001), 84.7% for P46 (*p* < 0.05), and 70.8% for P58 (*p* < 0.001) compared with controls. Invasion was also markedly decreased in KYSE30 cells after the ectopic expression of the P58 peptide aptamer (Fig. 4L, M, *p* < 0.0001). Thus, these in vitro findings suggested that ectopic expression of the P58 peptide aptamer, which was screened from the peptide aptamer library for targeting the interaction interface CDPΔC (101-1516) on CDP protein, inhibits proliferation, invasion, and migration in ESCC cells.

We then used parallel reaction monitoring-mass spectrometry (PRM-MS) to identify changes in some pivotal proteins in KYSE450 cells and thus to explore the mechanisms by which the P58 peptide aptamer influenced cellular function. As shown in Fig. 4N, there were clear alterations in the expression of specific proteins after the ectopic expression of the P58 peptide aptamer. Of these, the levels of proteins responsible for the maintenance of cell polarities and junctions, such as Scribble, Protein lin-7 homolog C (LIN7C), LLGL2, and Junctional adhesion molecule A (JAM1), were elevated, while those of proteins involved in cell proliferation, such as cyclin-dependent kinase 1 (CDK1), cyclin-dependent kinase 7 (CDK7), cyclin-dependent kinase 11A (CDK11A), antigen KI-67 (KI67), EGFR, tumor protein 63 (TP63), beta-catenin, PARP1, and cell cycle and apoptosis regulator 2 (CCAR2), were reduced. The level of E-cadherin protein increased, which may represent the induction of mesenchymal-to-epithelial transition (MET). On the other hand, the levels of several other proteins that promote apoptosis, such as P53 and BH3-interacting domain death agonist (BID), apoptosis-inducing factor mitochondria-associated 1 (AIFM1), and modulator of apoptosis 1 (MOAP1), increased (Supplementary PRM data, Data File S1). In addition, we also verified the changes of some pivotal proteins in KYSE450 cells after the ectopic expression of P58 peptide aptamer, and we found that the levels of E-cadherin and SLUG protein increased, whereas the levels of CDP and SOX2 protein decreased (Fig. 4O).

### P58 peptide aptamer slows tumor growth and metastasis in vivo

**Fig. 5:**
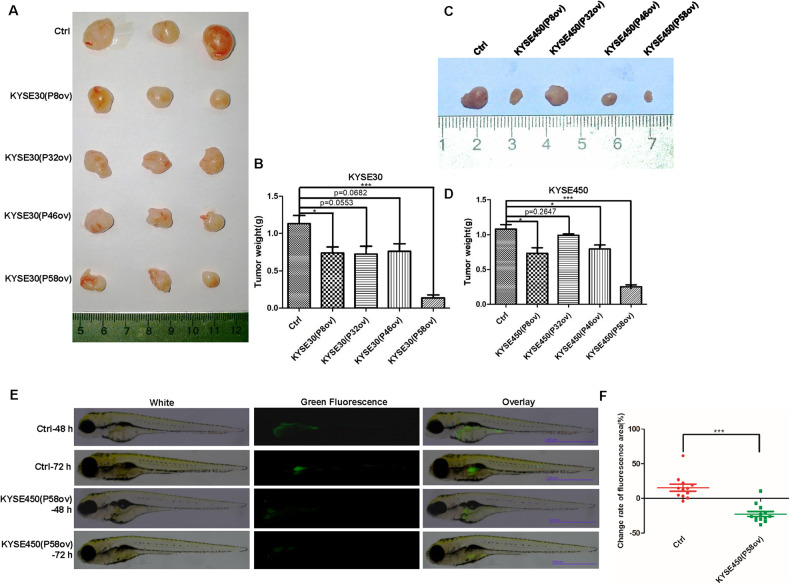
Ectopic expression of the P58 peptide aptamer in ESCC cells reduces tumor growth in nude mice and metastasis in zebrafish. **A** Xenograft tumors formed in nude mice after injection with KYSE30 control cells and peptide aptamer-transduced KYSE30 cells. **B** The P58 peptide aptamer slowed the growth of tumors formed by KYSE30 cells after statistical analysis (*p* < 0.001 for the P58 peptide aptamer and *p* < 0.05 for the P8 peptide aptamer). **C** Representative images of tumors formed in nude mice after injection with KYSE450 control cells and KYSE450 cells expressing P8, P32, P46, and P58 peptide aptamers. **D** The P58 peptide aptamer reduced the growth of tumors formed by KYSE450 cells after statistical analysis (*p* < 0.001 for the P58 peptide aptamer, *p* < 0.05 for the P8 and P46 peptide aptamers). **E** The P58 peptide aptamer reduced the metastasis of KYSE450 cells in zebrafish compared with the control peptide aptamer. Scale bar: 1.000 mm. **F** Statistical analysis of the change rate of the fluorescence area, which represents metastasis capability, after injection of KYSE450 cells expressing P58 peptide aptamer and control peptide aptamer (*p* < 0.001, *n* = 12). * *p* < 0.05, ** *p* < 0.01, *** *p* < 0.001 vs. control. The data represent the means ± SDs.

Mouse xenograft models were used to assess whether the peptide aptamers could reduce tumor growth in vivo. After the injection of KYSE30 cells expressing the P8, P32, P46, and P58 peptide aptamers and control cells into nude mice, it was found that tumor weights decreased to 65.5%, 64.2%, 67.5%, and 12.1% of controls after the ectopic expression of P8, P32, P46, and P58, respectively (Fig. 5 A, B, *p* < 0.05 for P8 and *p* < 0.001 for P58, *n* = 3). Similar results were also found with tumors derived from KYSE450 cells, and tumor weights were reduced to 67.8%, 91.8%, 73.6%, and 23.3% for P8, P32, P46, and P58, respectively, compared with controls (*p* < 0.05 for P8 and P46, *p* < 0.001 for P58) (Fig. 5C, D, *n* = 3). These findings indicate that the ectopic expression of the P58 peptide aptamer in ESCC cells achieved the optimal effect on reduced tumorigenesis in vivo.

To evaluate the effect of the P58 peptide aptamer on metastasis in vivo, the perivitelline spaces of zebrafish embryos were injected with 400 KYSE450 cells expressing the P58 peptide aptamer and the control. As shown in Fig. 5E, F, the change rate of the fluorescence area, representing the metastatic capability of cells, was decreased from 15.43% (mean value) in control cells to −22.53% (mean value) in P58 peptide aptamer-expressing cells (*p* < 0.001) (*n* = 12). These results suggest that the P58 peptide aptamer can significantly reduce the metastatic capability of ESCC cells in vivo.

### Synthetic peptide 58 suppresses tumor-associated phenotypes in ESCC cells

We next investigated the effects of adding peptide 58 directly to cultured ESCC cells. Peptide 58 and an empty control peptide were synthesized, both containing the cell-penetrating peptide YGRKKRRQRRR and the fluorophore TAMRA. Both peptides had a constrained conformation generated by a disulfide bond between two cysteines (supplied Fig. 3A).

**Fig. 6:**
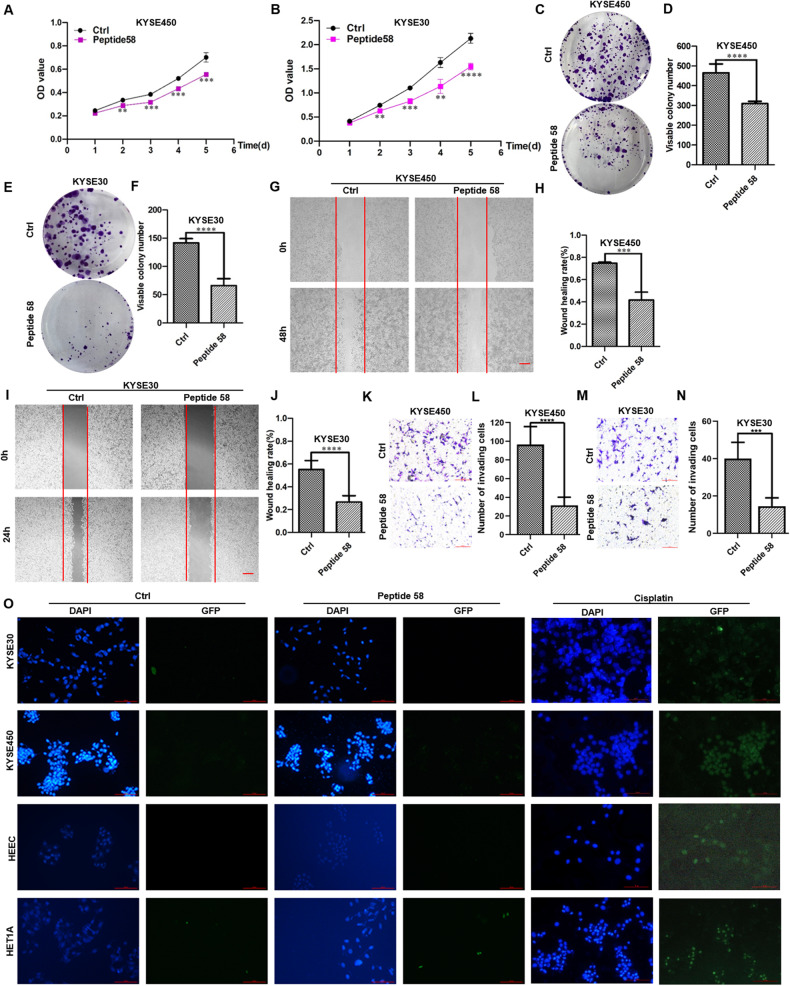
Synthetic peptide 58 inhibits multiple malignant processes of ESCC cells in vitro. **A** Reduced proliferation in KYSE450 cells occurred after treatment with peptide 58, as shown by the CCK8 assay (*p* < 0.001 on Days 3, 4, and 5). **B** Reduced proliferation in KYSE30 cells occurred after treatment with peptide 58, as shown by the CCK8 assay (*p* < 0.001 on Day 3 and *p* < 0.0001 on Day 5). **C**, **D** Decreased colony numbers in KYSE450 cells after peptide 58 treatment (*p* < 0.0001). **E**, **F** Decreased colony numbers in KYSE30 cells after peptide 58 treatment (*p* < 0.0001). **G**, **H** Attenuated migration in KYSE450 cells after treatment with peptide 58 (*p* < 0.001). **I**, **J** Attenuated migration in KYSE30 cells after treatment with peptide 58 (*p* < 0.0001). **K**, **L** Reduced invasion in KYSE450 cells after peptide 58 treatment (*p* < 0.0001). Scale bar: 100 μm. **M**, **N** Reduced invasion in KYSE30 cells after peptide 58 treatment (*p* < 0.001). Scale bar: 100 μm. **O** No obvious side effect on apoptosis was observed in several esophageal cell lines treated with peptide 58, the cells treated with cisplatin served as positive control, as shown by the TUNEL assay. Scale bar: 100 μm. * *p* < 0.05, ** *p* < 0.01, *** *p* < 0.001, **** *p* < 0.0001 vs. control. The data represent the means ± SDs.

To determine the appropriate test concentrations, the 50% inhibitory concentration (IC50) of peptide 58 was calculated using a CCK8 assay and statistical analysis after treatment for 48 h, and the IC50 values of peptide 58 in KYSE30 cells and KYSE450 cells were calculated to be 7.491 ng/μl and 15.23 ng/μl, respectively (Supplied Fig. 3B). The peptides were then added to the culture medium at 0.01 μg/μl, and various cellular activities were analyzed. Significantly reduced proliferation was observed in KYSE450 cells cultured with peptide 58, which was measured by the CCK8 assay after five days (Fig. 6A, *p* < 0.001). Similarly, peptide 58 also significantly inhibited KYSE30 cell proliferation on the fifth day (Fig. 6B, *p* < 0.0001). Additionally, peptide 58 also suppressed colony formation in both KYSE450 (Fig. 6C, D, *p* < 0.0001) and KYSE30 cells (Fig. 6E, F, *p* < 0.0001).

Investigation of the effects of peptide 58 on migration and invasion showed that it reduced migration significantly in KYSE450 cells (Fig. 6G), together with lowering the healing index from 74.8% (control peptide) to 41.7% (peptide 58) (Fig. 6H, *p* < 0.001). Similarly, peptide 58 also reduced migration in KYSE30 cells (Fig. 6I), and the healing index decreased from 55.1% (control peptide) to 26.6% (peptide 58) (Fig. 6J, *p* < 0.0001). Invasion, measured with transwell assays, was also inhibited in both KYSE450 cells (Fig. 6K, L, *p* < 0.0001) and KYSE30 cells (Fig. 6M, N, *p* < 0.001). Therefore, these results suggested that peptide 58 can suppress the proliferation, migration, and invasion of ESCC cells in vitro, consistent with the findings of ectopic expression of the P58 peptide aptamer. However, when the effects of the peptides on apoptosis were measured using the TUNEL assay, no significant side effects were observed. In contrast, apoptosis significantly occurred in these cells after treatment with cisplatin (Fig. 6O).

**Fig. 7:**
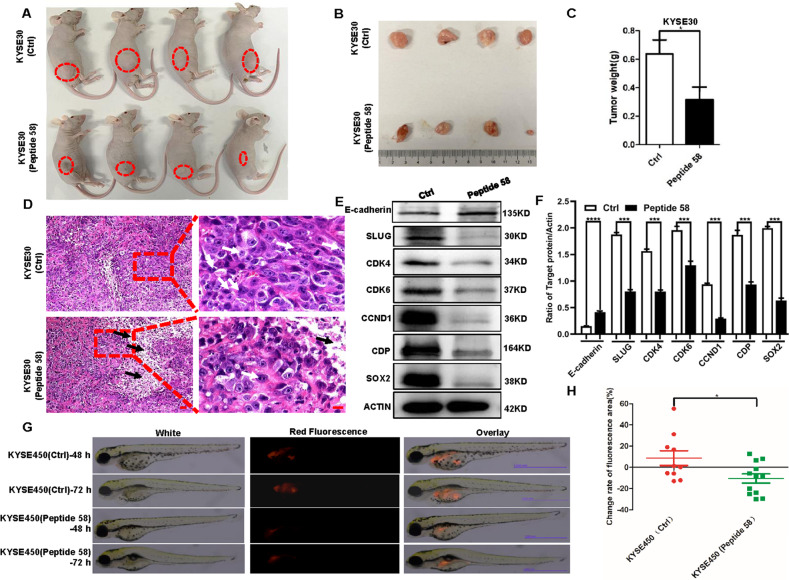
Peptide 58 slows the growth of KYSE30 cells in xenograft mice and reduces the metastasis of KYSE450 cells in zebrafish. **A**, **B** Tumors formed in nude mice after multiple rounds of injection with control peptide and peptide 58 (*n* = 4). **C** Reduced growth of KYSE30 cells was induced in nude mice after peptide 58 treatment (*p* < 0.05, *n* = 4). **D** Representative HE staining of tumors treated with control peptide and peptide 58. Note that the white arrows point to the dividing nucleus, and the black arrows point to the cells undergoing necrosis with karyolysis. Scale bar of left images: 30 μm. Scale bar of right images: 10 μm. **E** Changed levels of specific pivotal proteins in tumors after peptide 58 treatment. **F** Statistical analysis of altered protein densities induced by peptide 58. **G** Peptide 58 treatment significantly inhibited the metastasis of KYSE450 cells in zebrafish compared with the control. Scale bar: 1.000 mm. **H** Statistical analysis of the change rate of the fluorescence area, representing metastatic capability, after injection with KYSE450 cells treated with control peptide and peptide 58 (*p* < 0.05, *n* = 10). * *p* < 0.05, ** *p* < 0.01, *** *p* < 0.001 vs. control. The data represent the means ± SDs.

Xenograft mice with tumors formed by KYSE30 cells were injected with the control peptide and peptide 58 at concentrations of 0.04 μg/μl every two days, after which the mice were euthanized, and the tumors were harvested (Fig. 7A, B, *n* = 4). As shown in Fig. 7C, we found that the weights of tumors injected with peptide 58 were significantly lower than those of tumors injected with the control (*p* < 0.05), suggesting that peptide 58 can retard tumor growth. We also found differences between the tumor groups treated with control peptide and peptide 58. HE staining of harvested tumors suggested that there were higher rates of cell division in tumors treated with control peptide than those treated with peptide 58, while more cells exhibited necrosis with karyolysis after treatment with peptide 58 than with control peptide (Fig. 7D). To investigate which proteins were involved in this process, western blotting was performed with representative tumor tissue harvested from xenograft mice. The results showed that the levels of several key proteins, including CDK4, CDK6, CCND1, and SLUG, which are known as promoters of proliferation and invasion, were significantly lower in tumors treated with peptide 58. By contrast, the level of E-cadherin protein significantly increased in tumors treated with peptide 58. Intriguingly, we also observed significantly decreased levels of SOX2 and CDP proteins after peptide 58 treatment (Fig. 7E, F).

We also evaluated the effects of peptide 58 on metastasis using the zebrafish model. The change rates of the fluorescence area, which could represent the metastatic capability of KYSE450 cells after treatment with peptide, decreased from 8.80% in control cells to −14.39% in peptide 58-treated cells (Fig. 7G, H, *p* < 0.01, *n* = 10). This finding showed that peptide 58 significantly inhibited metastasis in KYSE450 cells in vivo. We next further compared the suppressive effects caused by peptide 58 and CUX1 knockdown. A significant effect on CUX1 knockdown was achieved after infection with pLK.O1 lentivirus expressing shRNA-1. Moreover, the suppressive effects of peptide 58 on ESCC cells were comparable to the effects caused by CUX1 knockdown, including proliferation, migration, and invasion (Supplied Fig. 4). In addition, we also compared the differences between KYSE450 cells caused by peptide 58 and SOX2 knockout. Interestingly, more significant decrease on cell proliferation, migration, and invasion were found in KYSE450 cells treated with peptide 58 than in KYSE450 (SOX2KO) cells (Supplied Fig. 5).

### Protein profiles of KYSE450 cells after peptide 58 treatment and bioinformatic analysis

**Fig. 8:**
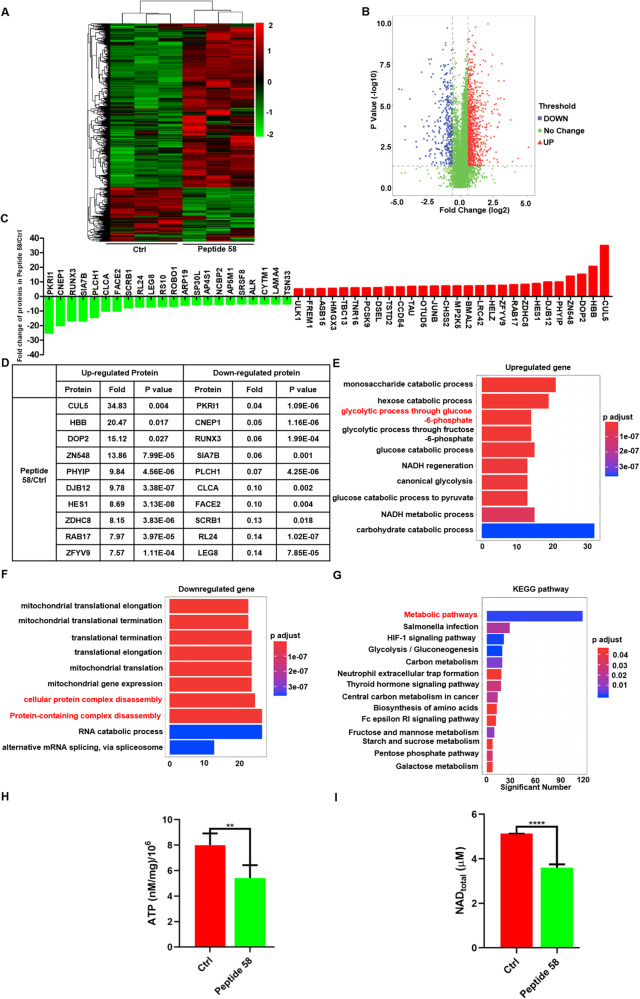
The underlying anticancer mechanism of peptide 58 is explored by proteomics, mass spectrometry, and DIA analysis. **A** Heatmap of differentially expressed proteins in KYSE450 cells upon peptide 58 treatment. **B** The volcano plot of proteins based on analysis of their fold changes. **C** Proteins with more than fivefold differential expression upon application of peptide 58 in KYSE450 cells. **D** Detailed information of the top 10 differentially expressed proteins upon application of peptide 58. **E** Biological process clustering analysis of upregulated proteins upon application of peptide 58. Note that some proteins were enriched in metabolic processes, and 14 proteins were enriched in glycolytic process through glucos-6-phosphate. **F** Biological process clustering analysis of downregulated proteins upon treatment with peptide 58. Note that some proteins are enriched in biological processes, including “cellular protein complex disassembly” and “protein-containing complex disassembly”. **G** KEGG pathway clustering analysis of changed proteins upon application of peptide 58. Note that 118 changed proteins are enriched in metabolic pathways. **H** Peptide 58 treatment significantly inhibited the ATP production in KYSE450 cells compared with the control (*p* < 0.01). **I** Peptide 58 treatment significantly suppressed the total NAD^+^/NADH in KYSE450 cells compared with the control (*p* < 0.0001).

To reveal the underlying mechanism of the suppressive effect on ESCC cells exerted by peptide 58, we performed proteomic and bioinformatic analyses. Proteomic analysis showed the upregulation of 4381 proteins and the downregulation of 1871 proteins in KYSE450 cells after culture with peptide 58, with the threshold value of the fold difference set at 1.5 (Fig. 8A, B). Of these proteins, 115 proteins were reduced more than twofold, while 389 proteins were increased more than twofold. Figure 8C shows the proteins with more than fivefold changes in expression, and the detailed information of the top 10 proteins is shown in Fig. 8D and Supplementary DIA data (Data File S2). Cullin-5 (CUL5) and PRKR-interacting protein 1 (PKRI1) were the most dramatically up- and downregulated proteins, respectively.

To identify the biological processes and cellular pathways influenced by peptide 58, clustering analysis based on biological processes and KEGG pathway analysis were also conducted. The results of the biological process clustering showed that “monosaccharide catabolic process”, “hexose catabolic process”, “glycolytic process through glucose-6-phosphate”, “glycolytic process through fructose-6-phosphate”, “glucose catabolic process”, “NADH regeneration”, “canonical glycolysis”, “glucose catabolic process to pyruvate”, “NADH metabolic process”, and “carbohydrate catabolic process” were the top 10 enriched processes using the upregulated proteins (Fig. 8E). “Mitochondrial translational elongation”, “mitochondrial translational termination”, “translational termination”, “translational elongation”, “mitochondrial translation”, “mitochondrial gene expression”, “cellular protein complex disassembly”, “protein-containing complex disassembly”, “RNA catabolic process”, and “alternative mRNA splicing, via spliceosome” were the top 10 enriched processes using the downregulated proteins (Fig. 8F). Notably, multiple downregulated proteins were involved in “cellular protein complex disassembly” and “protein-containing complex disassembly”, which is consistent with the finding that peptide 58 disrupted the SOX2/CDP protein complex.

KEGG-based clustering analysis showed enrichment in 14 pathways after treatment of KYSE450 cells with peptide 58, and metabolic pathways were enriched with 118 changed proteins. Seven pathways were closely associated with metabolism, suggesting that peptide 58 may suppress cancer progression by affecting metabolism, and CDP may closely correlate with metabolism (Fig. 8G). In conclusion, bioinformatic analysis using clustering based on biological processes and KEGG pathways indicated that many differences occurred after treatment of KYSE450 cells with peptide 58.

To verify the peptide 58 was involved in the glycolysis of KYSE450 cells, we detected the change of total ATP and NAD^+^/NADH in cells upon treatment with control peptide and peptide 58. Our result showed that ATP production dramatically decreased after treatment with peptide 58 when compared with control peptide (Fig. 8H, *p* < 0.01). In addition, the total NAD^+^/NADH, the molecules played a key role in regulating cellular metabolism and energy production, was significantly lower in group treated with peptide 58 than that in the control group (Fig. 8I, *p* < 0.0001). All these results suggested that peptide 58 may act as the inhibitor of the metabolism in ESCC cells.

## Discussion

SOX2 is necessary during organ development and the maintenance of stemness while also participating in tumor initiation, proliferation, migration, invasion, metastasis, resistance to therapy, and maintenance of stemness in malignant cells [3, 10, 14, 26]. SOX2 is thus a potential therapeutic target for cancer [27, 28]. SOX2 protein itself, acting as a transcription factor, has many binding sites on genomic DNA and exerts its influences on gene expression [29]. It also interacts with many proteins, such as CDK1 and PAPR1 [18, 30], suggesting that targeting SOX2 partners may also represent a useful therapeutic choice for cancer treatment [18, 31, 32]. Among these interacting proteins, CDP is a potential target, as it is documented to have oncogenic roles in cancer progression [20, 24, 33]. Indeed, we observed increased expression of CDP protein in ESCC tumor tissues in comparison with their matched controls, suggesting the potential therapeutic value of targeting CDP protein.

The use of peptide aptamers could be effective for the disruption of specific targets [34], and multiple peptide aptamers have been explored to inhibit target-specific proteins, such as cyclin-dependent kinase 2 (CDK2) [35], Nr13 protein [36], and mutant p53 [37], thereby inhibiting cancer progression. Here, we found that the SOX2 protein binds to the CDP protein and then identified the interaction interface using truncated mutations. After screening a peptide aptamer library for suitable aptamers, we identified peptide aptamer P58 as a competitive molecule. The peptide aptamer P58 was shown to inhibit a variety of tumorigenic processes in vitro and in vivo, such as proliferation, migration, invasion, tumor growth, and metastasis, while no side effects on proliferation and apoptosis were observed in noncancerous EPC2 and HEEC cells (Supplied Fig. 6). The PRM-MS and western blot results demonstrated P58’s suppressive roles on these malignant processes, as seen in the differential expression of some pivotal proteins, including E-cadherin and CDK7.

As protein complexes are known to play pivotal roles in multiple cellular activities, targeting their interactions or motifs may be an effective strategy for disrupting their activities [38]. Several protein complexes have been suggested as targets for cancer therapy, such as the GADD45beta/MKK7 complex [39] and the NOTCH transcription complex [40]. In addition to peptide aptamers, many other molecules, including peptides, have also been suggested as candidates for anticancer drug development due to their specific binding to the interaction motifs of some protein complexes. For example, the interaction between Twist and BRD4 plays roles in basal-like breast cancer, and this protein complex is required for WNT5A expression and malignant processes; the BET-specific inhibitors JQ1 and MS417 block both cancer cell invasion and tumorigenicity by targeting the interaction, thus representing a potential treatment for basal-like breast cancer [41]. Blocking the interaction between the NF-κB-regulated anti-apoptotic protein GADD45b and the JNK kinase MKK7 has been suggested for treating multiple myeloma, leading to the development of the DTP3 peptide, which can kill cancerous cells without affecting normal cells [39]. Similarly, the peptide SAHM1, targeting a critical binding site in the NOTCH transactivation complex, prevents NOTCH complex formation and the subsequent activation of downstream components, thereby providing a tool for T-cell acute lymphoblastic leukemia therapy [40]. Another peptide that disrupts the interaction between IRS1 and p110α E545K in colorectal cancer destabilized the p110α protein, reduced AKT phosphorylation, and slowed the growth of tumors expressing p110a E545K [42]. In addition, a peptide drug conjugate containing a heptapeptide (P7) has also been demonstrated to degrade HSP90 protein and induce apoptosis; thus, it should be effective for non-small cell lung cancer therapy [43]. All these studies suggest that the utilization of peptides for disrupting interactions between proteins or targeting specific proteins to suppress malignant processes is feasible. To date, many peptides have been used clinically, especially in cancer therapy, such as buserelin, goserelin, chlorotoxin, AN-152, dactinomycin, and lutetium Lu 177 dotatate [44].

In the present study, we used a synthetic peptide with the same amino acid sequence and structure as the P58 peptide aptamer, which was screened from a library, and observed its ability to reduce cancer progression in vitro and in vivo, consistent with the effects of the P58 peptide aptamer. Our synthetic peptides have a constrained conformation produced by a disulfide bond, and they also contain a peptide (YGRKKRRQRRR) to facilitate cell uptake and a fluorophore (TAMRA). After multiple rounds of injection with the control peptide and peptide 58 in tumor-bearing mice, we confirmed the therapeutic effectiveness of peptide 58 compared with the control. Peptide 58 treatment resulted in the changed expression of several pivotal proteins, such as CDK4, CDK6, CCND1, SLUG, and E-cadherin. Interestingly, we also observed that the levels of both SOX2 and CDP were significantly reduced, and we deduced that the formation of the SOX2/CDP protein complex may be helpful for strengthening the stability of these two proteins in ESCC cells.

To further dissect the mechanism underlying peptide 58’s actions in ESCC treatment, we analyzed protein changes using proteomics, mass spectrometry, and DIA analysis. We found that CUL5 and PKRI1 were the most upregulated and downregulated proteins among the changed proteins after treatment with peptide 58, respectively. CUL5 is an important component of Cullin-RING ligase-5 (CRL-5), which mediates the ubiquitylation and degradation of several key cellular proteins involved in cancers and viral infections. CUL5 is widely expressed in the body, including the esophagus, and appears to block the progression of small-cell lung cancer and gastric cancer [45, 46]. On the other hand, PKRI1 was the most downregulated protein and was reduced 25-fold after application of peptide 58, suggesting its close association with cancer and cancer progression. This result agrees with a previous study that found that PKRI1 is a driver gene on chromosome 7q for colorectal cancer and a factor of the spliceosome, and elevated expression of PKRI1 was significantly associated with unfavorable prognosis in patients with colorectal cancer (unpublished data provided by Yuki Oozato in the 79th annual meeting of the Japanese cancer association).

Taken together, these findings indicate that the SOX2/CDP protein complex may be an effective therapeutic target, and the small specific peptide 58, which targets the interacting interface on CDP protein, may be a candidate drug for treating ESCC. To achieve better therapeutic efficacy for ESCC, synthetic peptide 58 could be modified with other advanced technologies, such as nanomaterials, to specifically target and destroy malignant cells in ESCC patients.

## Methods

### Cell lines, mice, and reagents

The human ESCC cell lines KYSE30, KYSE450, TE-1 and TE-15 were obtained from ATCC (Manassas, VA, USA), and HEEC and HET1A, normal esophageal epithelial cell lines, were purchased from the Cell Bank of the Chinese Academy of Sciences. The EPC2 cell line was hTERT-immortalized with functionally intact p53 and p16 and maintained as previously described [3, 47]. KYSE30, KYSE450, HEEC, HET1A, TE-1 and TE-15 cells were cultured in RPMI medium (SH30809.01, HyClone, Beijing, China) or Dulbecco’s modified Eagle’s medium (DMEM; SH30022.01B; HyClone) supplemented with 10% fetal bovine serum (FBS; 10270-106; Gibco, Grand Island, NY, USA). Human ESCC biopsies were provided by Shanghai Outdo Biotech Company (HEso-Squ150CS-02), and the Medical Ethics Committee of Dongfang Hospital of Xiamen University approved all the experiments described herein. All 4-week-old male BALB/c nude mice were purchased from GemPharmatech Company (Nanjing, China) and were maintained in a specific-pathogen-free (SPF) facility. The xenograft study was performed in compliance with the Guide for the Care and Use of Laboratory Animals and was approved by the Institutional Animal Care and Use Committee of Dongfang Hospital, Xiamen University.

The FLAG Immunoprecipitation Kit (cat no: FLAGIPT1) was purchased from Sigma Aldrich (St. Louis, MO, USA). The antibodies included the following: anti-FLAG monoclonal antibody (M20008; Abmart, China); anti-SOX2 rabbit monoclonal (4900; Cell Signaling Technology, Danvers, MA, USA); anti-SOX2 rabbit polyclonal (WRAB-1236; Seven Hills, Cincinnati, OH, USA); anti-CDP rabbit polyclonal (ab73885; Abcam, Cambridge, UK); anti-E-Cadherin rabbit monoclonal (3195 s; Cell Signaling Technology); anti-SLUG rabbit monoclonal (9585 T; Cell Signaling Technology); anti-CDK4 rabbit monoclonal (12790; Cell Signaling Technology); anti-CDK6 rabbit polyclonal (AB20398a; BBI); anti-Cyclin D1 rabbit polyclonal (2922; Cell Signaling Technology); anti-beta Actin mouse monoclonal (AA128; Beyotime); anti-Ki67 rabbit monoclonal (12202; Cell Signaling Technology); HRP-conjugated goat anti-rabbit IgG (ab136817; Abcam); and HRP-conjugated goat anti-mouse IgG (ZB-2305; ZSGB-Bio, Beijing, China). Goat anti-rabbit IgG (H + L) superclonal^TM^ secondary antibody (A27034) and goat anti-rabbit IgG (H + L) superclonal^TM^ secondary antibody (A-11037) were obtained from Thermo Fisher (Waltham, MA, USA). Detailed information regarding the use of these antibodies is described in Supplied Table 4. Puromycin used for establishing stable cell lines was purchased from Santa Cruz Biotechnology (sc-108071; Dallas, TX, USA), and all other chemical reagents were purchased from Sigma Aldrich.

### Vector construction, lentivirus packaging, and stable cell line establishment

To obtain protein fused with VN173 or VC155 (kindly provided by Changdeng Hu from Purdue University), primers for the expression of SOX2 and CDP were designed (Supplied Table 2) and the constructs were verified by enzyme digestion and sequencing (Supplied Fig. 1A, B). To verify the interactions between SOX2 and CDP and between the interacting interface and the peptide aptamer candidates, constructs driving the expression of the FLAG-fused peptide were obtained after high-fidelity PCR with the primers listed in Supplied Table 3, enzyme digestion, ligation, and sequencing were then performed for verification (Supplied Fig. 1C, D). Lentiviral vectors expressing peptide aptamers were constructed, and lentivirus was obtained by cotransfection of the lentiviral constructs and packaging plasmids (Supplied Fig. 2A); stable cell lines were generated by lentivirus infection and puromycin selection (Supplied Fig. 2B).

To compare the suppressive effect caused by the synthetic peptide and gene knockdown, we synthesized two pairs of oligos to achieve knockdown of the CUX1 gene, and these two pairs of oligos are listed in Supplied Table 5. Then, these two pLKO.1 constructs (8453, Addgene) expressing shRNA were obtained after insertion of oligos. KYSE450 stable cell lines expressing these two shRNAs were established after lentivirus packaging, infection, and drug selection, respectively.

### BiFc assay for peptide aptamer screening

BiFc was used to verify the SOX2-CDP interaction interface, and peptide aptamers interacting with the interface were screened from a previously established aptamer library [25]. Constructs (0.2 μg) were cotransfected into HEK293T cells using lipofectamine™ 2000 (11668-019, Invitrogen, Waltham, MA, USA). The BiFc efficiency was assessed by cotransfection of 50 ng of pDsRed2-C1 encoding red fluorescent protein as an internal control. Cells were fixed with 4% paraformaldehyde and permeabilized in PBST (PBS with 0.5% Triton X-100). Nuclei were counterstained with DAPI in PBS, and the cells were examined and imaged under fluorescence microscopy (Nikon, Japan).

### Duolink proximity ligation assay

To detect the interaction between SOX2 and CDP, we used the Duolink® In Situ Proximity Ligation Assay kit (DUO92008, Sigma Aldrich). KYSE450 and KYSE30 cells were fixed in 4% paraformaldehyde for 10 min at room temperature, treated with 0.5% Triton X-100 for 5-10 min at room temperature, and blocked using 1× blocking solution for 60 min at 37 °C. Cells were then incubated with primary antibodies against SOX2 protein and CDP protein at 4 °C overnight, followed by washing with wash buffer A and incubation with PLA probes at 37 °C for 60 min. After washing two times with wash buffer A, the ligation-ligase solution was added and incubated at 37 °C for 60 min. Cells were then washed two times with wash buffer A and incubated with amplification-polymerase solution at 37 °C in the dark for 100 min. Finally, the cells were washed with wash buffer B and stained with mounting medium containing DAPI. Fluorescence images were captured under a confocal laser scanning microscope.

### Immunoprecipitation and western blotting

Constructs expressing the FLAG-fused SOX2 protein or peptide aptamer and the interaction interface on CDP were cotransfected into HEK293T cells with polyethylenimine; cells transfected with only one construct served as the control. After 48 h, the cells were immunoprecipitated using the FLAG Immunoprecipitation Kit according to the provided protocol, and anti-FLAG and anti-CDP antibodies were used to identify the respective protein bands.

### Histological analysis and immunofluorescence

These analyses were performed as previously described [3, 14].

### Peptide design and synthesis

Peptide synthesis was performed by Genscript Co., Ltd. (Nanjing, China), with a penetrating signal sequence and the fluorophore TAMRA included at the N-terminus (Supplied Fig. 3A). The peptides were resuspended to 2 mg/ml in DMSO and stored at −20 °C.

### Cancer cell function assays

In vitro assays for cell proliferation, colony formation, wound healing, and invasion through Matrigel and in vivo assays for tumor growth and metastasis were performed; detailed protocols are provided in our previous studies [3, 14, 25]. For the in vivo assay, briefly, KYSE30 or KYSE450 cells expressing the peptide aptamer 58 or control combined with Matrigel were injected subcutaneously into the flanks of the nude mice, these mice were treated with CO_2_ for anaesthesia and the tumors were excised after eight weeks. To investigate the antitumor activity of peptide 58, 0.04 μg/μl of peptide 58 or the control peptide was injected into tumors derived from KYSE30 cells every two days, and the tumors were analyzed after 4 weeks.

### Metastasis studies using a zebrafish model

To investigate the effects of the P58 aptamer and peptide 58 on metastasis, fertilized AB zebrafish eggs were developed for 48 hours, and the perivitelline space of each embryo was injected with 400 KYSE450 cells expressing the P58 peptide aptamer and control or KYSE450 cells treated with 0.01 μg/μl peptide 58 and control peptide for 48 h. After injection into the perivitelline space of embryos in zebrafish eggs, the zebrafishes were cultured, and images of the fluorescence area in zebrafishes were obtained under stereoscopic microscopy. Fluorescence images were acquired using Capture software at 48 h and 72 h. ImageJ was used to measure the fluorescence area. The metastatic capability was reflected by the change rate of the fluorescence area, and the change rate of the fluorescence area was calculated and analyzed using the formula: (S_72_ − S_48_)/S_48_ × 100%, where S_72_ is the area 72 h after injection, and S_48_ is the area 48 h after injection. The Mann‒Whitney *U* test was used to assess differences between these groups, with *P* < 0.05 considered significant.

### Protein sample preparation, LC‒MS/MS, and bioinformatic analyses

Cells were lysed in lysis buffer containing 100 mM Tris-HCl (pH 8.0), 1% SDS, 8 M urea, 20 mM DTT, and protease inhibitors for 15 min on ice, followed by centrifugation for 10 min at 3000 × *g*/4 °C. The total protein concentration in the supernatant was measured using the BCA method. Proteins were then reduced (50 mM DTT, 60 min, 37 °C), alkylated (20 mM iodoacetamide, 30 min, room temperature, in the dark), diluted with 1.5 M urea and digested with trypsin (sequencing grade, Promega) at a 1:50 (enzyme: substrate) ratio for 8 h at 37 °C. The digestion was quenched with 1% TFA, and the peptides were desalted with a desalting column (Thermo Fisher) before drying with vacuum centrifugation.

The peptides were resuspended in 20 µl buffer A (0.1% TFA), and 6 µl peptides were analyzed by nanoflow liquid chromatography on an Easy nLC1000 online coupled to an Orbitrap Fusion Lumos Tribrid mass spectrometer through a nanoelectrospray flex-ion source (all Thermo Fisher). The samples were applied to a precolumn (Acclaim PepMap C18, 100 μm × 2 cm, Thermo Fisher) at a flow rate of 3 μl/min and then separated on an analysis column (Acclaim PepMap C18, 75 μm × 15 cm, Thermo Fisher) using a flow rate of 0.3 μl/min. Peptides were separated and directly electrosprayed into the mass spectrometer using the linear gradient: 0-3 min, 4-7% B; 3-59 min, 7-20% B; 59-80 min, 20-30% B; 80-82 min, 30-90% B.

Data were acquired using DIA mode. A full MS scan was acquired by analyzing 200-2000 m/z at resolution 120,000 (at m/z of 200) in the Orbitrap using an AGC target value of 4.8E5 and maximum IT of 50 ms. After the MS scan, twenty MS/MS scans were then obtained, each having a 30,000 resolution at a m/z of 200, AGC target 2E5, and collision energy of 32%, with the default charge state set to 2 and the maximum IT set to 72 ms. The cycles of the 20 MS/MS scans (center of isolation window) with three types of wide isolation windows were as follows (m/z): 368.5, 399, 418, 432, 444.5, 455, 464.5, 473.5, 482.5, 491, 499.5, 508, 516.5, 525, 533, 541.5, 550, 558, 566.5, 575.5. The full cycle of MS and MS/MS scan acquisition was approximately 3 s in duration and was repeated throughout the LC/MS/MS analysis. Bioinformatic analyses, specifically Gene Ontology (GO) annotation, KEGG annotation and enrichment, domain annotation and analysis, and subcellular localization analysis, were undertaken. Proteomic data have been deposited at PRIDE and are publicly available as of the date of publication (accession number PXD035683).

### ATP production assay

The ATP levels in KYSE450 cells were determined using an Enhanced ATP Assay Kit (S0027, Beyotime Biotechnology), and the assay was performed according to the manufacturer’s guidance. Cells treated with control peptide and peptide 58 were lysed and then centrifuged for 5 min at 4 °C and 12,000*g*, and their supernatants were obtained, respectively. Before ATP detection, 100 μL detecting solution was put into the 1.5 mL Eppendorf tube and incubated for 5 min at room temperature. The 20 μL supernatant was then added to the tube, mixed quickly, and read within 30 min using luminometer. Total ATP levels were calculated from the luminescence signals and were normalized by the protein concentrations.

### NAD^+^/NADH assay

The cellular content of NAD^+^ and NADH was determined using a NAD^+^/NADH Assay Kit with WST-8 (S0175, Beyotime Biotechnology) according to the manufacturer’s instructions. Briefly, 1 × 10^6^ KYSE450 cells were plated into each well of six-well plates and incubated with 200 μL of NAD^+^/NADH extraction solution. Repeated pipetting to lyse the cells and then centrifuged for 5 min at 12,000*g* and 4 °C. 20 μL supernatant was taken into the 96-well plate, add working solution and chromogenic solution in turn. After incubation at 37 °C without light, the value of absorbance at 450 nm was detected, and the concentration was calculated according to the NADH standard curve. The total NAD^+^/NADH was calculated and normalized by the protein concentrations.

### Statistical analysis

Data were from a minimum of three independent experiments from at least three separate isolations or collections and are represented as the means ± SDs. Differences between groups were compared using repeated-measures ANOVA, and all analyses were conducted using GraphPad PRISM. V8.0 software. Unpaired *t* tests were used to determine the significance of differences between two groups, with *P* < 0.05 considered significant.

### Supplementary information

Supplementary Figures

Supplementary Tables

Data file S1

Data file S2

Original Data File

## Data Availability

The authors declare that all data generated or analyzed during this study are included in this published article. The data that support the findings of this study are available from the corresponding author upon reasonable request.
