# Supplementary material for: Targeting the SOX2/CDP protein complex with a peptide suppresses the malignant progression of esophageal squamous cell carcinoma
Source: Cell Death Discov. 2023 Oct 27;9:399. doi: 10.1038/s41420-023-01693-7 (PMC10611744; doi:10.1038/s41420-023-01693-7)
Supplement: Supplementary file 1 — Supplementary Figures [file 41420_2023_1693_MOESM1_ESM.docx]

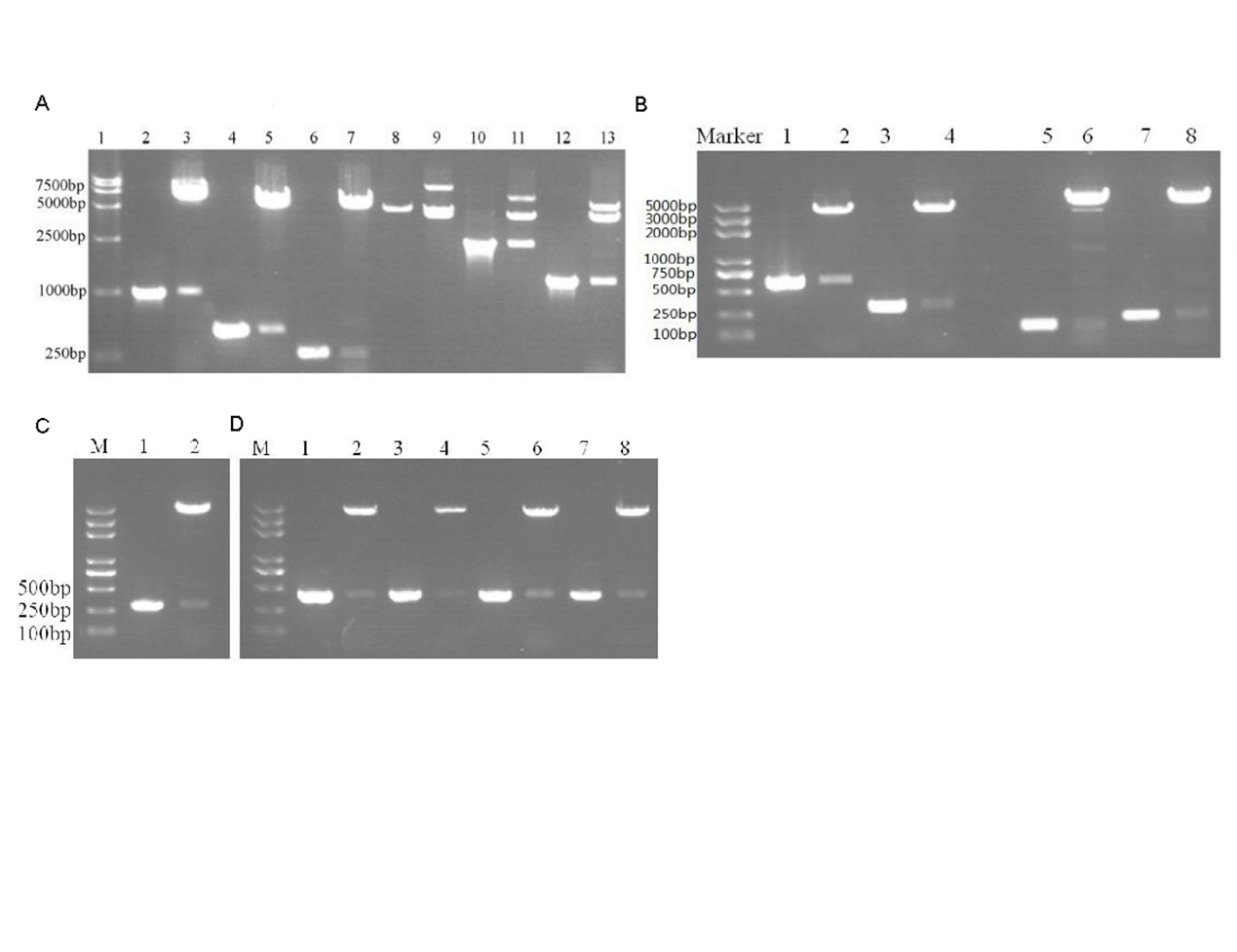


**Supplied Figure 1. Construction of vectors for BiFc assay and vectors for screening and validating interaction between CDP∆C (101-1516) and peptide aptamers.** The primers used for the construction of these vectors are showed in Supplementary table S2. To obtain the pBiFcVN173-SOX2 for expressing the full-length of SOX2 protein and its mutants expressing truncated SOX2 protein, a plasmid containing the SOX2 coding sequence was amplified with high fidelity PCR. The PCR products were subcloned into the pBiFcVN173 digested with EcoRI and XbaI, generating pBiFcVN173-SOX2 and their truncated mutants. To obtain the pBiFcVC155-CDP for expressing the full-length of CDP protein and its mutants expressing truncated CDP protein, a plasmid containing the CDP coding sequence was amplified with high fidelity PCR. The PCR products were subcloned into the pBiFcVC155 digested with SalⅠ and KpnI, generating pBiFcVC155-CDP and their truncated mutants.

(**A**) Lane 1 is DNA Marker, lane 2、4、6 represent PCR product of the full-length of SOX2, SOX2ΔN (1-167) and SOX2ΔN (1-237), and lane 3, 5, 7 represent the product of pBiFcVN173-SOX2 and their mutants digested with EcoRI and XbaI, respectively. Lane 8, 10, 12 represent PCR product of the full-length of CDP, CDPΔC (759-1516) and CDPΔC (401-1516), and lane 9, 11, 13 represent the product of pBiFcVC155-CDP and their mutants digested with SalⅠ and KpnI. (**B**) lane 1, 3 represent PCR product of CDPΔC (201-1516) and CDPΔC (101-1516), and lane 2, 4 represent the product of pBiFcVC155-CDPΔC (201-1516) and pBiFcVC155-CDPΔC (101-1516) digested with SalⅠ and KpnI, respectively. Lane 5, 7 represent PCR product of SOX2ΔN (1-277) and SOX2ΔN (1-257), and lane 6, 8 represent the product of pBiFcVN173- SOX2ΔN (1-277) and pBiFcVN173- SOX2ΔN (1-257) digested with EcoRI and XbaI. (**C**) lane 1 represents the PCR product of CDP∆C (101-1516), which is the interaction interface on CDP protein, lane 2 represents the product of pBiFcVN173-CDP∆C (101-1516) digested with EcoRI and XbaI. (**D**) lane 1, 3, 5 and 7 represent the PCR product of fragment expressing peptide aptamer P8, P32, P46 and P58, respectively. While lane 2, 4, 6 and 8 represent the product of pCMV-Tag2B-peptide, which drives the expression of FLAG-fused peptide aptamers, digested with BamHI and EcoRI.


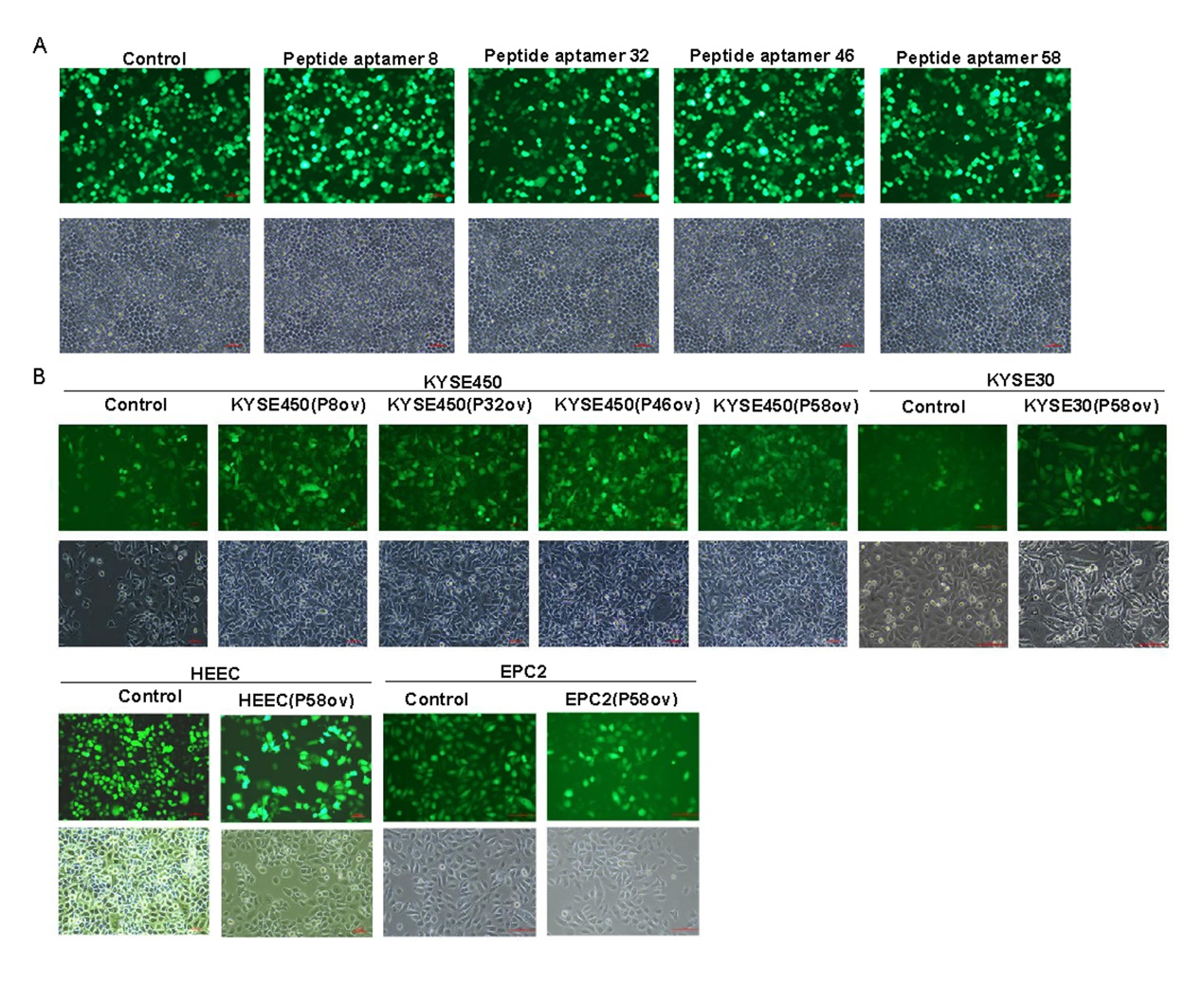


**Supplied Figure 2.** **Lentivirus packaging and the establishment of stable cell lines expressing peptide aptamers.** (**A**) After transfection of pCDH-CMV-peptide aptamer-IRES-GFP-EF1-Puro with VSVG and PHR into HEK293T cells, lentivirus was successfully produced and harvested. (**B**) Stable expression of various peptide aptamers in different cell lines following lentiviral infection and drug selection. Scale bar: 100μm.


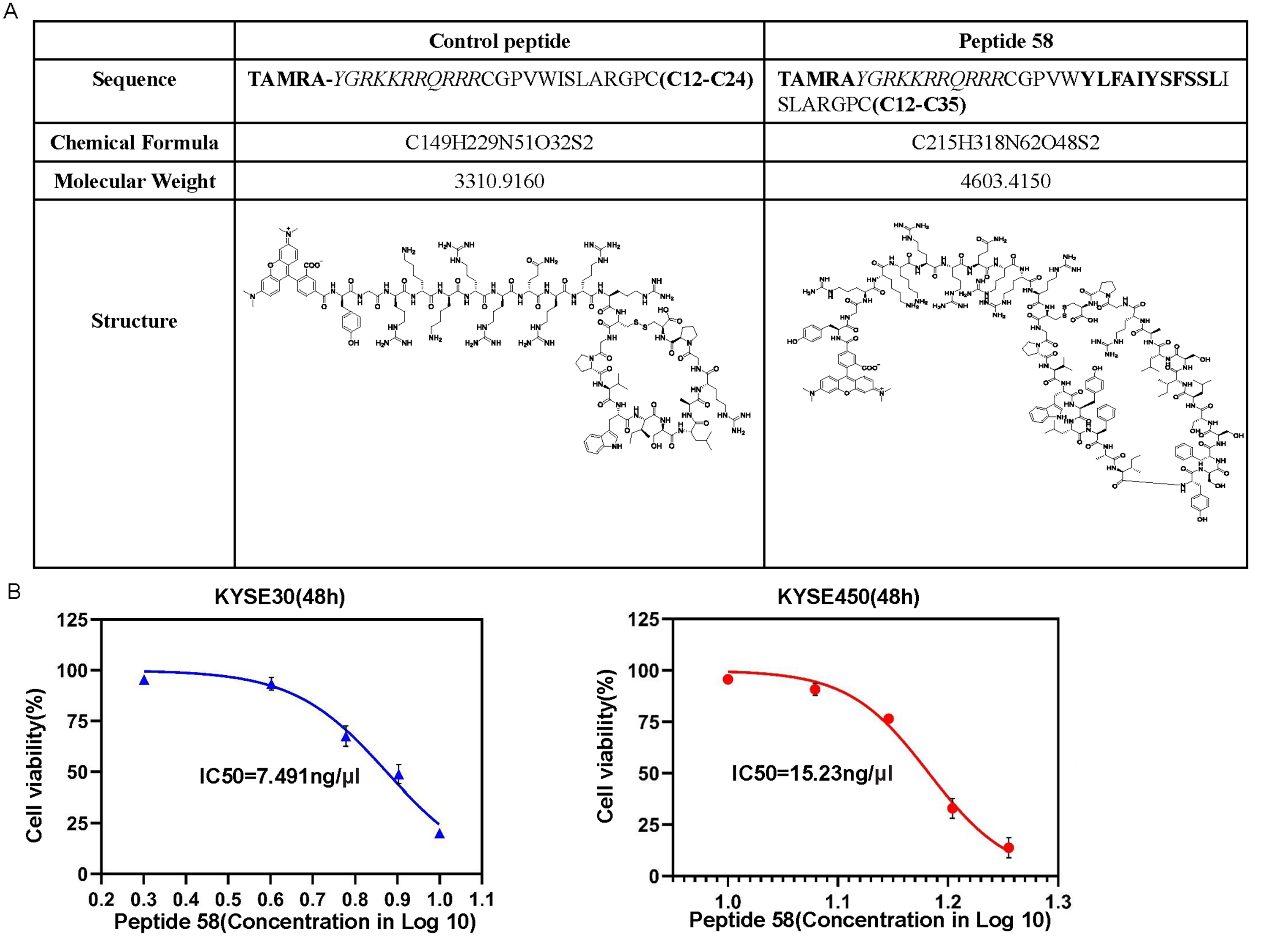


**Supplied Figure 3. Detailed information for synthetic peptides and IC50 determination.** (**A**) The detailed information of chemically synthetic control peptide and peptide 58. (**B**) The IC50 of peptide 58 in KYSE30 cells and KYSE450 cells were determined by CCK8 assay and statistical analysis.


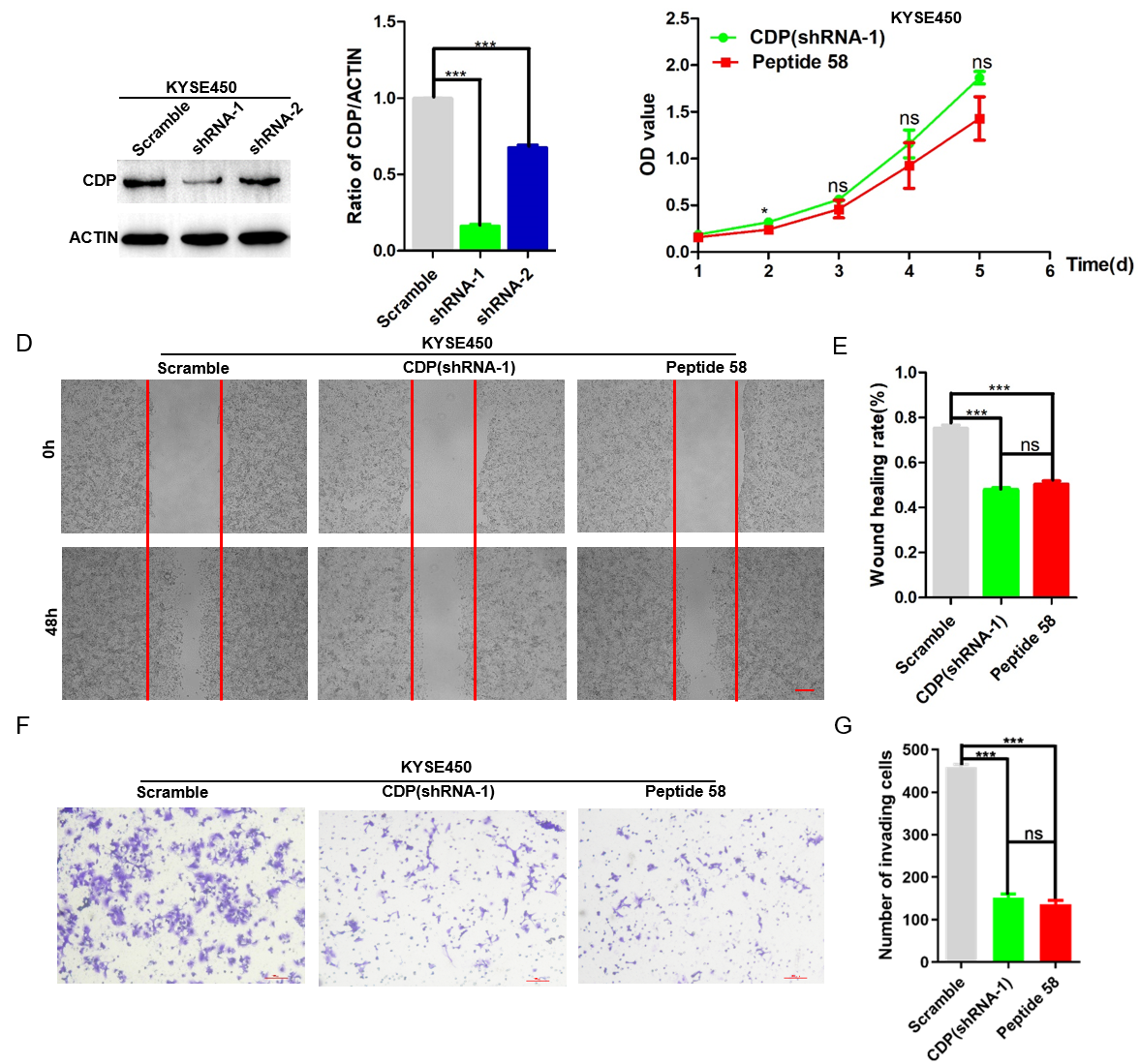


**Supplied Figure 4. The suppressive effects of peptide 58 on ESCC cells are comparable to the effects caused by CUX1 knockdown, including proliferation, migration, and invasion.** (**A, B**) Significant effect on CUX1 knockdown was achieved in KYSE450 cells after infection with lentivirus expressing shRNA-1 (p<0.001 for shRNA-1 and shRNA-2). (**C**) no significant difference on cell proliferation occurred between KYSE450 cells expressing shRNA-1 and KYSE450 cells treated with peptide 58 (p>0.05). (**D, E**) no significant difference on cell migration happened between KYSE450 cells expressing shRNA-1 and KYSE450 cells treated with peptide 58. (**F, G**) no significant difference on cell invasion existed between KYSE450 cells expressing shRNA-1 and KYSE450 cells treated with peptide 58 (p>0.05). Scale bar: 100μm. * p<0.05, ** p<0.01, *** p<0.001 vs. control. The data represent the means ± SDs.


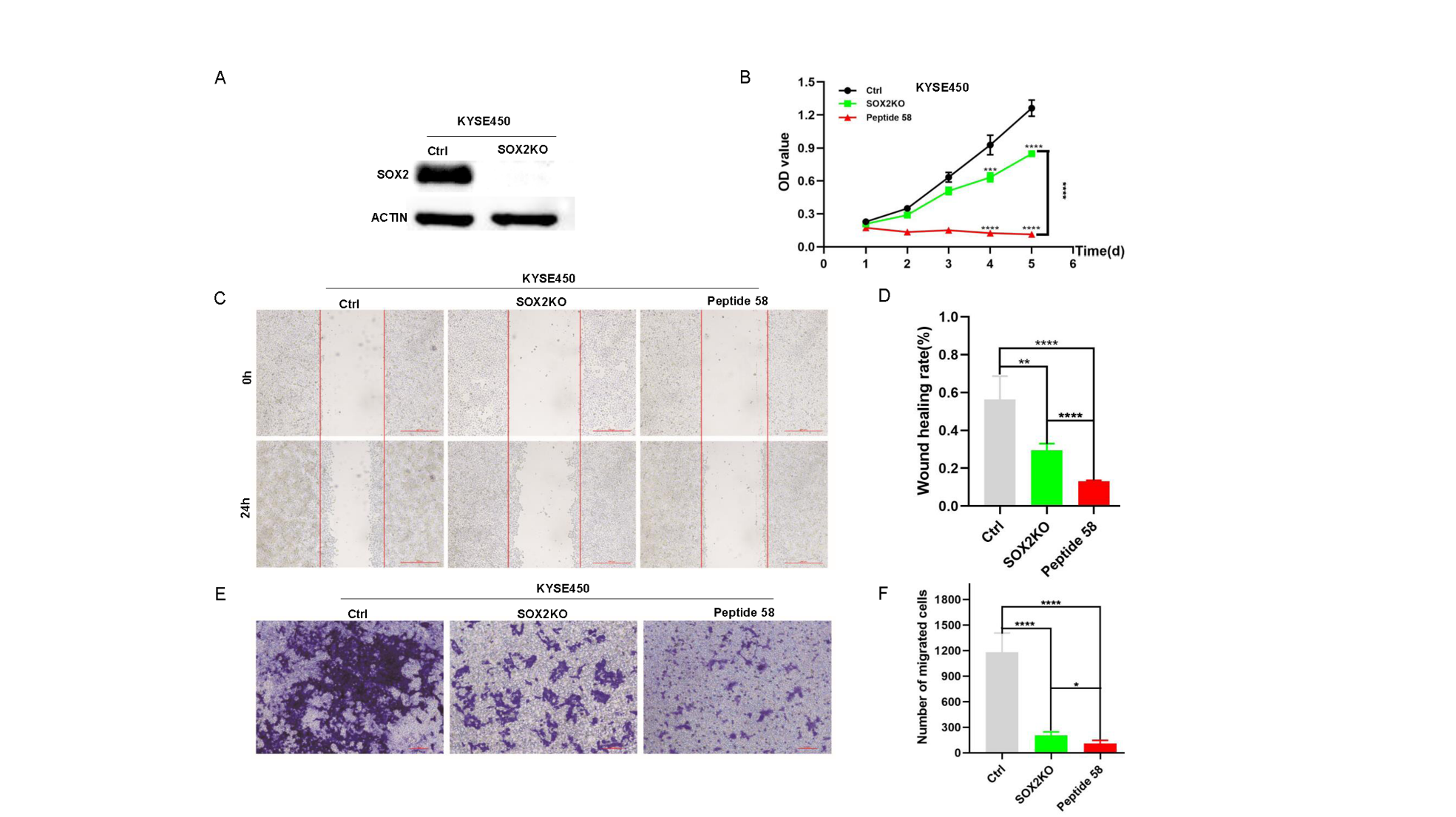


**Supplied Figure 5. The peptide 58 and SOX2 knockout reduce the proliferation, migration, and invasion of ESCC cells.** (**A**) Significant suppressive effect on SOX2 protein level was obtained in KYSE450 cells after stable expression of sgRNA. (**B**) More significant decrease on cell proliferation occurred in KYSE450 cells treated with peptide 58 than in KYSE450 (SOX2KO) cells (p<0.0001). (**C, D**) More significant decline on cell migration happened in KYSE450 cells treated with peptide 58 than in KYSE450 (SOX2KO) cells (p<0.0001). (**E, F**) More significant reduction on cell invasion existed in KYSE450 cells treated with peptide 58 than in KYSE450 (SOX2KO) cells (p<0.05). Scale bar: 100μm. * p<0.05, ** p<0.01, *** p<0.001, **** p<0.0001 vs. control. The data represent the means ± SDs.


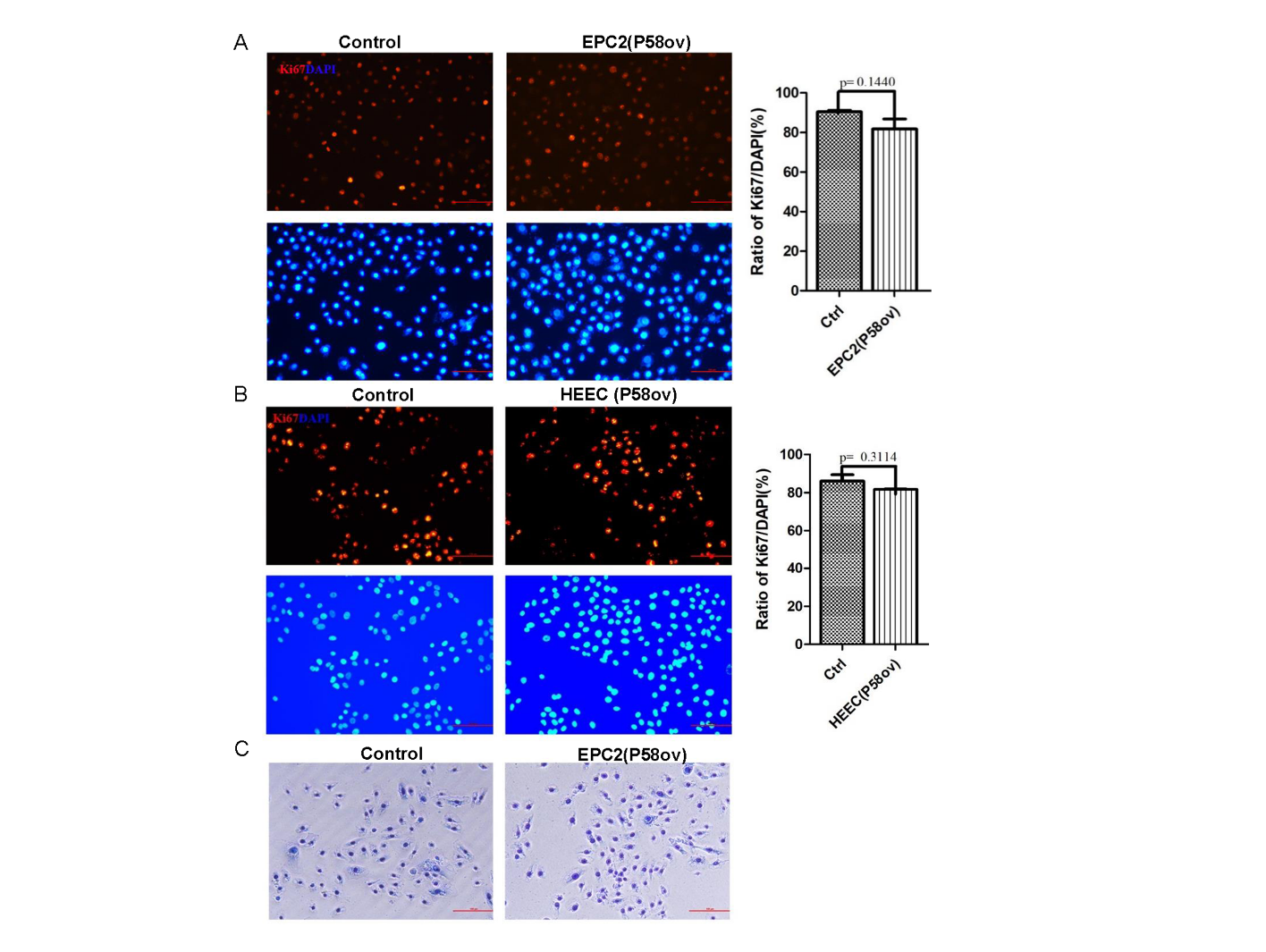


**Supplied Figure 6. Ectopic expression of P58 peptide aptamer does not affect the proliferation and apoptosis of esophageal epithelial cell lines (EPC2 and HEEC).** (**A, B**) No side effect on cell proliferation occurred after the ectopic expression of P58 peptide aptamer. (**C**) No side effect on cell apoptosis occurred after the ectopic expression of P58 peptide aptamer. Note apoptotic cells are rarely observed in EPC2 control cells and EPC2 cells expressing peptide aptamer P58. Scale bar: 100μm.
