# Supplementary material for: Targeting the SOX2/CDP protein complex with a peptide suppresses the malignant progression of esophageal squamous cell carcinoma
Source: Cell Death Discov. 2023 Oct 27;9:399. doi: 10.1038/s41420-023-01693-7 (PMC10611744; doi:10.1038/s41420-023-01693-7)
Supplement: Supplementary file 2 — Supplementary Tables [file 41420_2023_1693_MOESM2_ESM.docx]

**supplied Table 1. Clinicopathological characteristics of 75 ESCC patients used for measuring CDP protein levels**

|  | | **Cases in each group** | **Percent** |
| --- | --- | --- | --- |
| **Histopathological grade** | **I** | **11** | **14.7%** |
|  | **I-II** | **14** | **18.7%** |
|  | **II** | **34** | **45.3%** |
|  | **II-III** | **5** | **6.7%** |
|  | **III** | **11** | **14.7%** |
| **TNM Stage** | **T Stage** | **24** | **32.0%** |
|  | **N Stage** | **46** | **61.3%** |
|  | **M Stage** | **5** | **6.7%** |

**supplied Table 2. Primers used for determining interaction domain between SOX2 and CDP protein**

| **Target product** | **Primer sequence (5’- 3’)** | **Product size** |
| --- | --- | --- |
| pBiFc-VN173-SOX2 | Forward:5’-CGGAATTCAATGTACAACATGATGGAGAC-3’  Reverse:5’-TGCTCTAGACATGTGTGAGAGGGGCAG-3’ | 1000bp |
| pBiFc-VN173-SOX2  ΔN (1-167) | Forward:5’-CGGAATTCAAACGGCAGCTACAGCATGA-3’  Reverse:5’-TGCTCTAGACATGTGTGAGAGGGGCAG-3’ | 500bp |
| pBiFc-VN173-SOX2  ΔN (1-237) | Forward:5’-CGGAATTCAGGCTCCATGGGTTCGGTG-3’  Reverse:5’-TGCTCTAGACATGTGTGAGAGGGGCAG-3’ | 250bp |
| pBiFc-VN173-SOX2  ΔN (1-257) | Forward:5’-CGGAATTCATCCTCCCACTCCAGGGCG-3’  Reverse:5’-TGCTCTAGACATGTGTGAGAGGGGCAG-3’ | 180bp |
| pBiFc-VN173-SOX2  ΔN (1-277) | Forward:5’-CGGAATTCACTCCCCGGCGCCGAGGT-3’  Reverse:5’-TGCTCTAGACATGTGTGAGAGGGGCAG-3’ | 120bp |
| pBiFc-VC155-CDP | Forward:5’-ACGCGTCGACCATGGCGGCCAATGTGGGAT-3’  Reverse:5’-GGGGTACCGAACTCCCATTCGATAGGT-3’ | 5000bp |
| pBiFc-VC155-CDP  ΔC (759-1516) | Forward:5’-ACGCGTCGACCATGGCGGCCAATGTGGGAT-3’  Reverse:5’-GGGGTACCGGACAGAAGCTTGGGGGT-3’ | 2500bp |
| pBiFc-VC155-CDP  ΔC (401-1516) | Forward:5’-ACGCGTCGACCATGGCGGCCAATGTGGGAT-3’  Reverse:5’-GGGGTACCGTTCTTCTCCAGCAACAGCAC-3’ | 1200bp |
| pBiFc-VC155-CDP  ΔC (201-1516) | Forward:5’-ACGCGTCGACCATGGCGGCCAATGTGGGAT-3’  Reverse:5’-GGGGTACCCTTTGAGGTGGTGGACATCT-3’ | 600bp |
| pBiFc-VC155-CDP  ΔC (101-1516) | Forward:5’-ACGCGTCGACCATGGCGGCCAATGTGGGAT-3’  Reverse:5’-GGGGTACCTGGGACGTCAATCAATCTTTT-3’ | 300bp |

**supplied Table 3. Primers used for screening and validating peptide aptamer against the interaction domain on CDP protein**

| **Target product** | **Primer sequence (5’- 3’)** | **Product size** |
| --- | --- | --- |
| pBiFc-VN173-CDP  ∆C (101-1516) | Forward:5’-CGGAATTCAATGGCGGCCAATGTGGGAT-3’  Reverse:5’-TGCTCTAGATGGGACGTCAATCAATCTTTT-3’ | 300bp |
| pCMV-Tag2B-peptide | Forward:5’-CGGGATCCATGAGCGATAAAATTATTCAC-3’  Reverse:5’-CGGAATTCCAGGTTAGCGTCGAGGAA-3’ | 330bp |

**supplied Table 4. Information of antibodies used for Western blot (WB), immunostaining (IF) and immunochemistry (IHC)**

| **Name of antibody** | **Vendor & category number** | **Dilution** |
| --- | --- | --- |
| SOX2 | Seven Hills WRAB-1236 | 1:2000 (WB) |
| SOX2 | Abcam ab79351 | 1: 200 (IF) |
| CDP | Abcam ab73885 | 1:500 (WB)/1:500 (IHC) /1:100 (IF) |
| E-cadherin | Cell Signaling 3195s | 1:1000 (WB) |
| SLUG | Cell Signaling 9585T | 1:1000 (WB) |
| CDK4 | Cell Signaling 12790 | 1:1000 (WB) |
| CDK6 | BBI AB20398a | 1:300 (WB) |
| CCND1 | Cell Signaling 2922 | 1:1000 (WB) |
| Beta-Actin | Beyotime AA128 | 1:2000 (WB) |
| Ki67 | Cell Signaling 12202 | 1:200 (IF) |
| Anti-FLAG monoclonal antibody | Abmart M20008 | 1:5000 (WB) |
| Goat anti-Mouse HRP | ZSGB-BIO ZB-2305 | 1:5000 |
| Goat anti-Rabbit HRP | Abcam ab136817 | 1:2000 |
| Goat anti-rabbit IgG (H+L) superclonal^TM^ secondary antibody | ThermoFisher SCIENTIFIC A27034 | 1:1000 |
| Goat anti-Rabbit IgG(H+L) Sencondary Antibody, Alexa-fluor 594 | ThermoFisher SCIENTIFIC A11037 | 1:1000 |

**supplied Table 5. Oligos used to express two different shRNAs for CUX1 knockdown**

| **shRNA name** | **Oligo sequence (5’- 3’)** |
| --- | --- |
| shRNA -1 | Forward:5’-CCGGGCACGATATTGAAACAGAGAACTCGAGTTCTCTGTTTCAATATCGTGCTTTTTG-3’  Reverse:5’- AATTCAAAAAGCACGATATTGAAACAGAGAACTCGAGTTCTCTGTTTCAATATCGTGC-3’ |
| shRNA -2 | Forward:5’-CCGGCGAAACCATAGCTCTTGAGAACTCGAGTTCTCAAGAGCTATGGTTTCGTTTTTG-3’  Reverse:5’- AATTCAAAAACGAAACCATAGCTCTTGAGAACTCGAGTTCTCAAGAGCTATGGTTTCG-3’ |
