# Supplementary material for: Targeting the SOX2/CDP protein complex with a peptide suppresses the malignant progression of esophageal squamous cell carcinoma
Source: Cell Death Discov. 2023 Oct 27;9:399. doi: 10.1038/s41420-023-01693-7 (PMC10611744; doi:10.1038/s41420-023-01693-7)

Images in Figure 1G

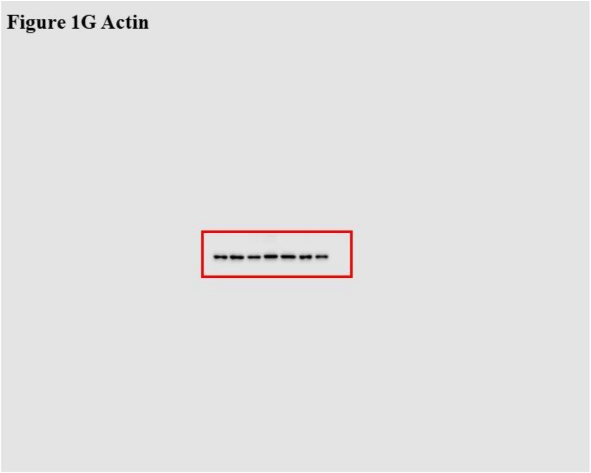

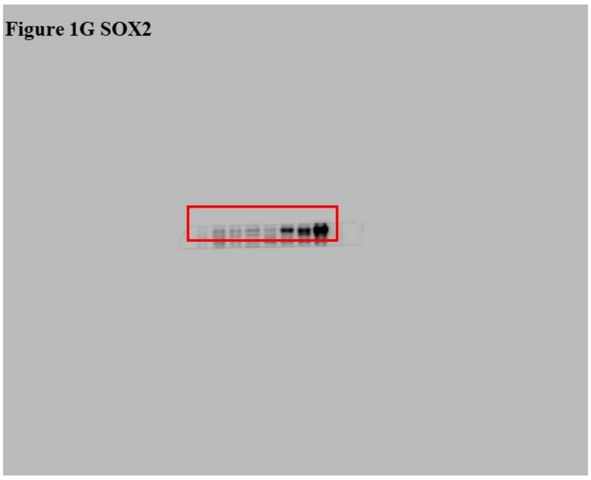

Figure 1G CDP

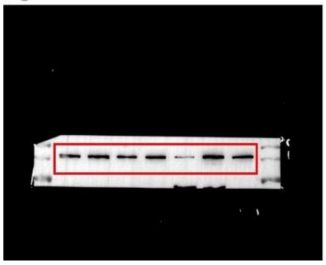

Images in Figure 2E

Images in Figure 2E CDP

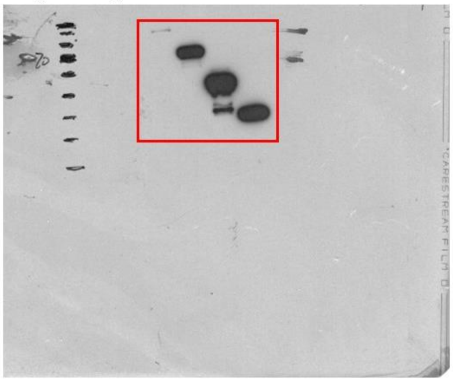

Images in Figure 2E SOX2

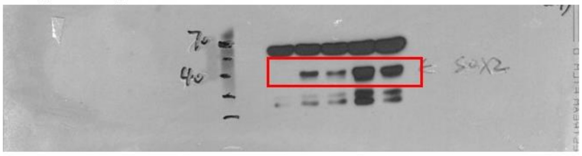

Images in Figure 3B

Images in Figure 3B CDP antibody

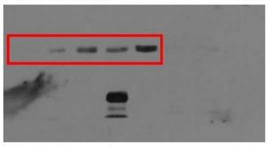

Images in Figure 3B FLAG antibody

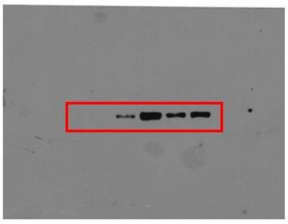

Images in Figure 4O

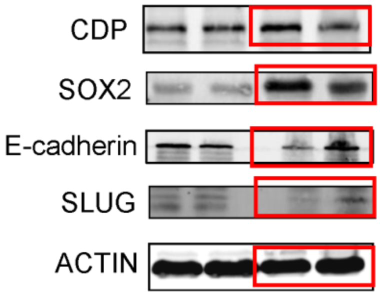

Images in Figure 7E

Images in Figure 7E SLUG

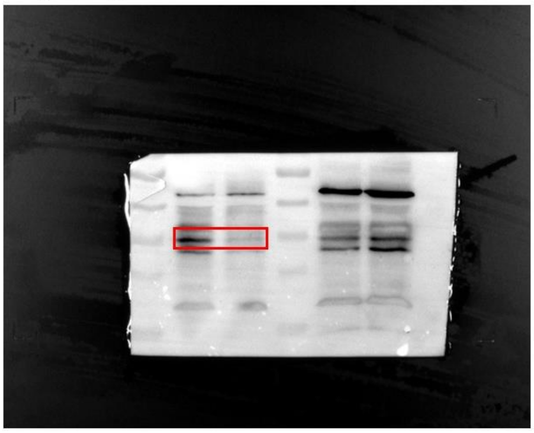

Images in Figure 7E CDK4

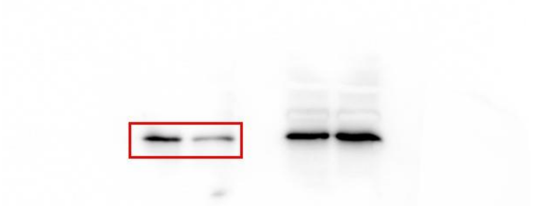

Images in Figure 7E CDK6

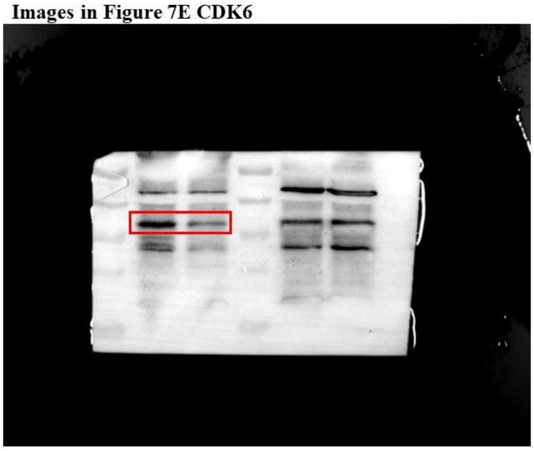

Images in Figure 7E CCND1

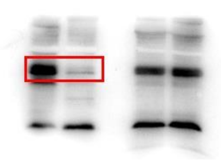

Images in Figure 7E CDP

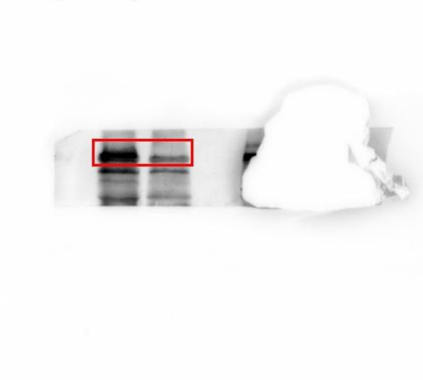

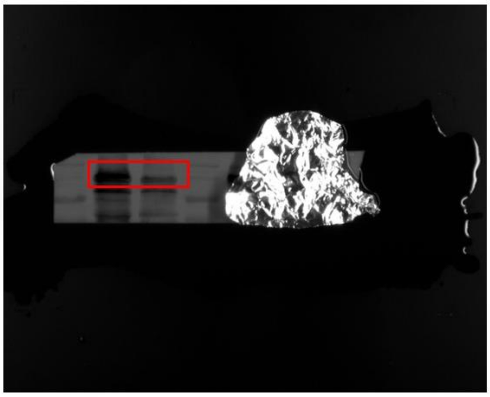

Images in Figure 7E SOX2

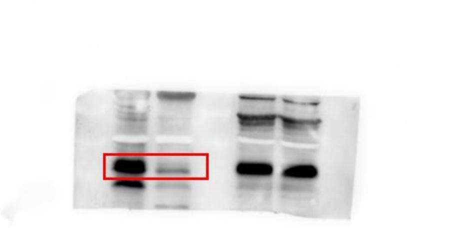

Images in Figure 7E ACTIN

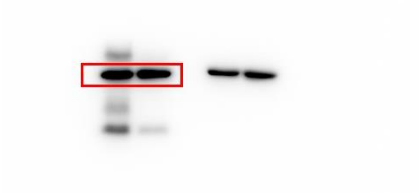

Supplement: Supplementary file 5 — Original Data File [file 41420_2023_1693_MOESM5_ESM.pdf]
